# Engineered K1F bacteriophages kill intracellular *Escherichia coli* K1 in human epithelial cells

**DOI:** 10.1038/s41598-018-35859-6

**Published:** 2018-12-03

**Authors:** Christian Møller-Olsen, Siu Fung Stanley Ho, Ranti Dev Shukla, Tamas Feher, Antonia P. Sagona

**Affiliations:** 1 School of Life Sciences, University of Warwick, Gibbet Hill Road, CV4 7AL Coventry, UK; 2 Synthetic and Systems Biology Unit, Biological Research Centre of the Hungarian Academy of Sciences, Szeged, Hungary; 3 Doctoral School in Biology, Faculty of Science and Informatics, University of Szeged, Szeged, Hungary

## Abstract

Bacterial infections can be treated with bacteriophages that show great specificity towards their bacterial host and can be genetically modified for different applications. However, whether and how bacteriophages can kill intracellular bacteria in human cells remains elusive. Here, using CRISPR/Cas selection, we have engineered a fluorescent bacteriophage specific for *E. coli* K1, a nosocomial pathogen responsible for urinary tract infections, neonatal meningitis and sepsis. By confocal and live microscopy, we show that engineered bacteriophages K1F-GFP and *E. coli* EV36-RFP bacteria displaying the K1 capsule, enter human cells via phagocytosis. Importantly, we show that bacteriophage K1F-GFP efficiently kills intracellular *E. coli* EV36-RFP in T24 human urinary bladder epithelial cells. Finally, we provide evidence that bacteria and bacteriophages are degraded by LC3-associated phagocytosis and xenophagy.

## Introduction

Bacteriophages or phages are viruses that infect bacteria and are the most abundant organisms on earth^1^. Bacteriophages present significant diversity and play an important role in the evolution of their host^2^. Bacteriophages have also been found inside the human body^3–6^ and more recently have been shown to enter via different mechanisms in human cells^7,8^. Bacteriophages are used to treat bacterial infections (phage therapy) and the interest in phage therapy has grown increasingly in recent years due to the emerging problem of antibiotic resistance of many bacterial pathogens^9–12^. Due to the recent advances of molecular and synthetic biology, bacteriophages can be easily genetically modified to obtain preferable characteristics for different applications^13,14^. A recently established efficient strategy applies the CRISPR/Cas System for the selection of recombinant bacteriophages^15–17^. This has opened new perspectives for phage therapy, by making genetically modified bacteriophages more easily attainable^18^. Such an approach could provide solutions to naturally resistant nosocomial bacterial pathogens, such as *Escherichia coli* K1. *E. coli* K1 is a gram-negative pathogen, responsible for a wide range of diseases, including sepsis, neonatal meningitis, urinary tract infections and inflammatory bowel syndrome^19–21^.

The virulence of *E. coli* K1 (*E. coli* O18:K1:H7) is attributed to its K1 polysaccharide capsule. The K1 capsular polysaccharide (K1 antigen) is an α-2-8-linked homopolymer of sialic acid (NeuNAc), which is responsible for the virulence and pathogenicity of these strains. The K1 antigen acts as a natural antiphagocytic barrier for the bacteria^22^. In addition, structural similarities between K1 and human tissue components indicate that immune tolerance may also be a factor of capsular *E. coli* pathogenesis^23^. Due to these characteristics, *E. coli* O18:K1:H7 is able to invade human endothelial and epithelial cells and cause the corresponding diseases referred to above^21,24–26^.

Phage K1F is a T7-like phage, which was first isolated in sewage and specifically infects *E. coli* strains that such as *E. coli* O18:K1:H7^27^. Despite its similarities to phage T7 at the genome scale, phage K1F incorporates the endosialidase enzyme within its tail structure instead of the T7 tail fiber protein, which enables the attachment to- and degradation of the K1 polysaccharide capsule^27^. The K1 polysaccharide capsule has therefore been shown to be a barrier to T7^28^ and a receptor for phage K1F.

Here we have developed a novel *in vitro* model system for studying phage therapy for *E. coli* K1 in T24 human urinary bladder epithelial cells. We have applied the CRISPR/Cas system to engineer fluorescent phage K1F that are able to infect the *E. coli* EV36 strain, an *E. coli* K12/K1 hybrid derivative with the ability to display a Kl polysaccharide capsule morphologically similar to that of *E. coli* K1 clinical isolates^29^. This system has enabled us to observe that both bacteria and bacteriophages invade T24 cells and that phage K1F kill intracellular *E. coli* EV36. We also show that upon being phagocytosed, bacteria and phages are degraded via different pathways: phage K1F is degraded mostly via LC3-assisted phagocytosis, whereas *E. coli* EV36 strain activates xenophagy. In the presence of both bacteria and bacteriophages, xenophagy is activated, indicating that the pathogen is sufficient to activate autophagy, whereas the bacteriophages on their own cannot activate autophagy to the same extent.

## Results

### Phage K1F targets *E. coli* EV36-RFP strain

We initially confirmed the specificity of phage K1F for *E. coli* cells displaying a K1 capsule. For our experiments, we used the *E. coli* EV36 strain, an *E. coli* K-12-K1 hybrid derivative with the ability to express a Kl polysaccharide capsule morphologically similar to that of *E. coli* K1 clinical isolates^29^. We first compared in liquid culture the specificity of phage K1F and phage T7 for targeting the K1 capsule of the *E. coli* EV36 strain (Suppl. Fig. S1A left panel). By measuring the optical density at 600 nm over a period of 3.5 hours, we observed a drop in optical density in *E. coli* EV36 liquid culture only upon phage K1F addition. When no phage was added or when phage T7 was added, the *E. coli* EV36 bacteria continued to grow, indicating that solely phage K1F was capable of lysing them. We further tested phage K1F specificity towards an *E. coli* K-12 strain (MG1655 cells) (Suppl. Fig. S1A right panel) and we observed that in the presence of phage K1F or when no phage was added, *E. coli* MG1655 bacteria continued to grow and only upon T7 phage addition did the optical density of the liquid culture drop, indicating that T7 is specific to K-12 strains but not phage K1F. These results were further confirmed by plaque assays (Suppl. Fig. S1B). The phage K1F selected from a single plaque was further purified with a CsCl column and we performed transmission electron microscopy to reveal the exact pattern and structure of phage K1F (Suppl. Fig. S1C). This purified phage K1F was used for all our phage-related experiments and was the basis of the genetically engineered fluorescent phage K1F. In order to make *E. coli* EV36 bacteria easily visualized by microscopy, we transformed them with an RFP-expressing plasmid to provide them with a red colour (Suppl. Fig. S1D). Upon successfully obtaining *E. coli* EV36-RFP bacteria, we tested both in liquid culture (Suppl. Fig. S1E left panel) and with plaque assays (Suppl. Fig. S1E right panel) the ability of phage K1F to efficiently kill *E. coli* EV36-RFP. Our results showed that expression of RFP in *E. coli* EV36 did not seem to affect phage K1F’s ability to infect this strain, when compared with empty *E. coli* EV36 (Suppl. Fig. S1E left panel). All together our results confirm that phage K1F targets *E. coli* EV36-RFP.

### Genome engineering of fluorescent phage K1F-GFP followed by CRISPR/Cas selection

**Figure 1:**
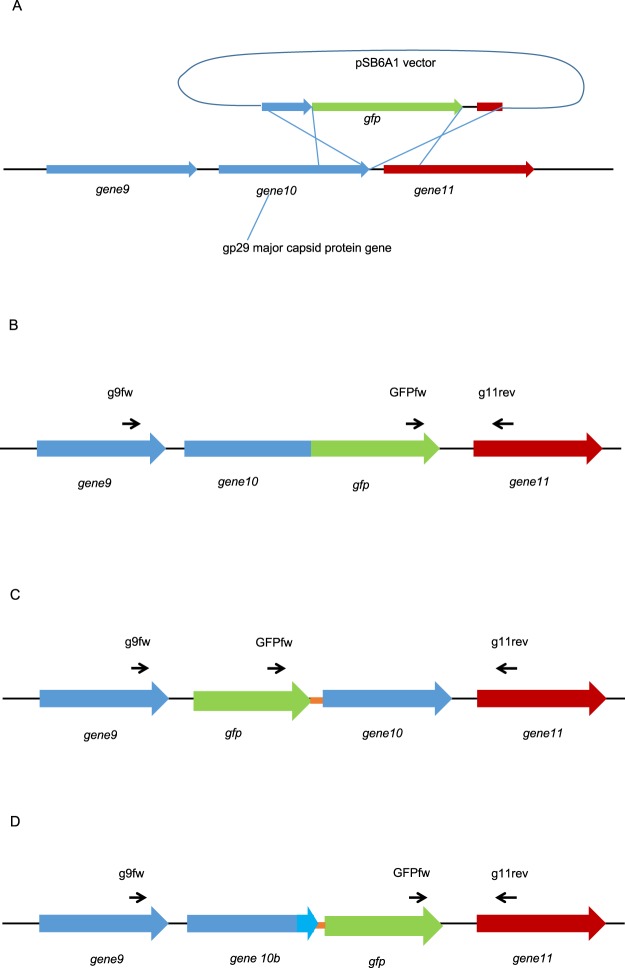
Construction of fluorescent phages K1F. (**A**) The engineering of three different constructs was attempted using *in vivo* recombination between a plasmid-borne donor DNA and *gene10* of the phage, encoding the major and minor capsid proteins. The double crossover required for recombination is shown for the process generating the C-terminal GFP fusion of the major capsid protein. (**B–D**) The expected results of the three engineering strategies are displayed: *g10::gfp*, the C-terminal GFP fusion of the major capsid protein-encoding gene (**B**), *gfp::g10*, the N-terminal GFP fusion of the major capsid protein-encoding gene (**C**) and *g10b::gfp*, the C-terminal GFP fusion of the minor capsid protein-encoding gene, i.e. *gene10b* (**D**). Linker peptide-encoding sequences used for the two latter constructs are depicted by orange lines. Thin arrows represent PCR primers used for screening and validation of recombinant phages.

To engineer a fluorescent phage K1F, we decided to integrate the superfolder green fluorescent protein (GFP) gene into the phage’s genome. Our primary aim was to generate translational fusions of GFP with *gene10* (or *g10*), which encodes the major capsid protein gp29. It is worth noting, that *gene10* can undergo a −1 translational frameshift in its 3′ region, resulting in the addition of 44 triplets. The protein encoded by this extended ORF (referred to as *gene10b* or *g10b*) functions as the minor capsid protein of the virion. For phage genome editing, we relied on the strategy of providing the donor DNA on a plasmid residing in the bacterial strain used to grow the targeted phage on (Fig. 1A), combined with the CRISPR/Cas selection of recombinant phages (Suppl. Fig. S2A,B)^16,17^. In our case, the strain hosting the donor DNA was *E. coli* EV36^29^. The donor DNA cassette, consisting of the GFP gene flanked by appropriate homology arms, was cloned into the pSB6A1 plasmid. We attempted the engineering of three different recombinant phages: phage K1F*g10::gfp* would carry the *gfp* gene fused to the 3′ end of *gene10*, making a C-terminal GFP fusion of the major capsid protein (Fig. 1A,B); analogously, phage K1F*gfp::g10* would express the N-terminal GFP fusion of the major capsid protein (Fig. 1C) and phage K1F*g10b::gfp* would harbour the *gfp* gene fused to the 3′ end of *gene10b*, making a C-terminal GFP fusion of the minor capsid protein (Fig. 1D). Plasmid and phage constructs are summarized in Table S3.

**Figure 2:**
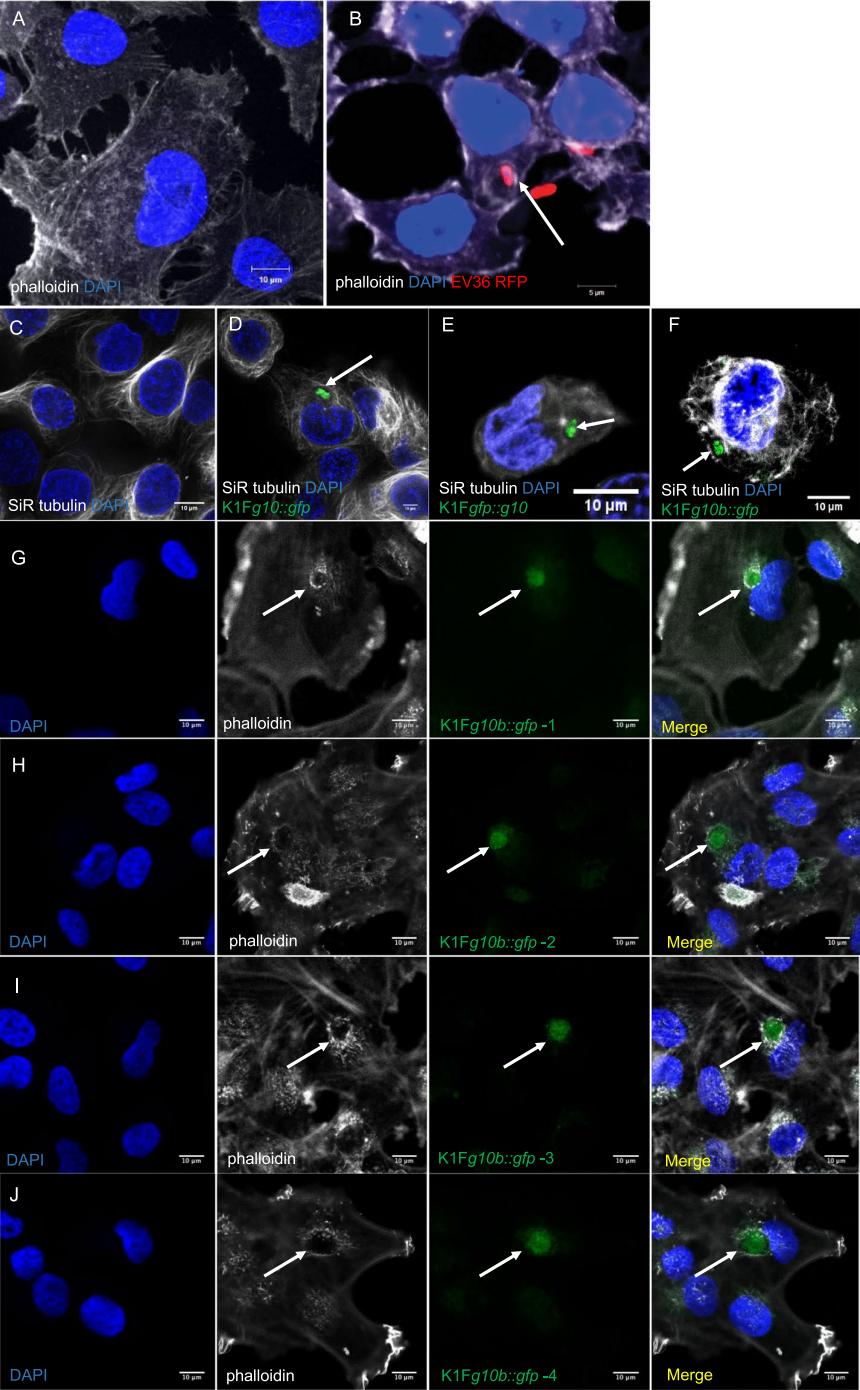
Image analysis of *E. coli* EV36-RFP bacteria and phage K1F-GFP constructs inside epithelial human cells. (**A,B**) Fluorescent images showing human epithelial T24 cells alone (**A**) and infected with *E. coli* EV36-RFP (**B**). DAPI stain is shown in blue. Phalloidin stain is shown in grey. (**C–F**) Fluorescent images showing human epithelial T24 cells alone (**C**) and infected with different constructs of fluorescent phages K1F: *g10::gfp* (**D**), *gfp::g10* (**E**), *g10b::gfp* (**F**). Arrows annotate the location of fluorescent phage K1F accumulation in vacuoles. DAPI stain is shown in blue. SiR-tubulin stain is shown in grey. (**G–J**) Fluorescent images showing T24 cells infected with the four pure fluorescent *g10b::gfp* phages K1F upon *in vivo* CRISPR/Cas selection (Suppl. Fig. S2D). Arrows annotate the location of pure fluorescent *g10b::gfp* phages K1F accumulation in vacuoles. DAPI stain is shown in blue. Phalloidin is shown in grey.

Growing phage K1F on *E. coli* EV36 carrying either of the three donor DNA molecules (Table S1) permitted the generation of phage mixtures containing both wild-type and recombinant phages. The presence of recombinants was indeed PCR-verified in each phage lysate using the GFPfw-g11rev primer pair (Suppl. Fig. S2C). However, the PCR-screening of plaques generated from these phage mixes did not result in the identification of recombinant plaques, thereby prohibiting the isolation of pure recombinant phage suspensions. To enrich recombinant phages within the phage mix, we applied CRISPR/Cas selection by growing the phage mix on *E. coli* EV 36 harbouring various pCas9 plasmid derivatives. The CRISPR/Cas9 machineries on these selection plasmids were engineered to cleave the wild-type *gene10*, but leave the *gfp*-fusion construct intact (Suppl. Fig. S2A,B). CRISPR/Cas selection of phages K1F*g10::gfp* using the plasmid pCas9g10 however, did not yield recombinant plaques, nor did it increase the fraction of recombinants within the mix (data not shown). Next, the CRISPR/Cas selection process was applied to the phage mix of phages K1F*g10b::gfp*. We used two different selection plasmids: pCas9K1FC1 and pCas9K1FC2, differing only in the encoded crRNA spacer sequences. After two rounds of growth of the phage mix on the two selection strains, recombinant plaques were observed with a frequency of 4/20, but only when using pCas9K1FC2 for selection. Phages retrieved from the positive plaques were further grown on *E. coli* EV36 cells and exposed to plaque assays to re-test for GFPfw-g11rev PCR fragment positivity. For all the four phage lysates, 5/5 plaques displayed the correct PCR product, verifying that the four original plaques of phages K1F*g10b::gfp* were homogenous and that the C-terminal minor capsid gene fusions were stably maintained in the phage genomes. PCR analysis with primers g9fw-g11rev from all the four recombinant plaques yielded a single product of 2591 bp, as opposed to the 1831 bp obtained from phage K1F (Suppl. Fig. S2D), again supporting successful *gfp* integration and the clonal nature of the phages K1F*g10b::gfp*. Sequencing the g9fw-g11rev region also verified correct integration, apart from a C to T transition at 22829, leading to Thr to Ile change in the C terminal region of the minor capsid protein. Each of the four phage K1F*g10b::gfp* lines underwent fluorescent microscopic analysis (Fig. 2G,H,I and J). The stable fluorescent phages K1F (K1F*g10b::gfp*) were used in the downstream experiments to detect phage K1F localisation in human cells.

### K1-displaying *E. coli* EV36-RFP bacteria and engineered phage K1F-GFP invade T24 human urinary bladder epithelial cells

We next examined whether engineered phage K1F-GFP (approximately 1 × 10^8^ PFU/ml) and *E. coli* EV36-RFP bacteria (approximately 2 × 10^7^ CFU/ml) can invade T24 human urinary bladder epithelial cells. It has been shown previously that *E. coli* 018:K1:H7 can enter T24 cells via microtubule and microfilament dependent pathways^24^. We infected T24 cells with *E. coli* EV36-RFP and stained them with phalloidin (for the actin cytoskeleton) and DAPI (for the nucleus) (Fig. 2A,B). Using confocal microscopy, we observed that *E. coli* EV36-RFP could invade T24 cells. This was further confirmed with live time-lapse microscopy (Supplementary Fig. S7A). We then stained T24 cells with SiR-tubulin, a live cell microtubule probe and NucBlue Live ReadyProbes for nuclear staining in live cells (Fig. 2C) and we further incubated with C-terminal major capsid protein-labelled phage K1F-GFP mix (K1F*g10::gfp*) (Fig. 2D), N-terminal major capsid protein-labelled phage GFP-K1F mix (K1F*gfp::g10*) (Fig. 2E) and C-terminal minor capsid protein-labelled pure phage K1F-GFP (K1F*g10b::gfp*) (Fig. 2F). In all the above cases, it was shown that phage K1F-GFP invaded T24 cells and localised inside vacuoles. Additionally, it was further confirmed by confocal microscopy (Suppl. Fig. S6A,B) and live time-lapse microscopy (Suppl. Fig. S7B), that phage K1F-GFP co-localised with *E. coli* EV36-RFP (Suppl. Fig. S6B) with great specificity (Suppl. Fig. S6A), indicating that the phage can target the bacteria in a human cell environment. We then continued analysing *in vivo* in more detail the pattern and localisation of the CRISPR-Cas selected four fractions (Suppl. Fig. S2D) of stable phage K1F*g10b::gfp* (K1F*g10b::gfp*-1 to -4, Fig. 2G–J). It was clearly shown that all the fractions of stable phage K1F*g10b::gfp* could invade T24 cells and localise inside vacuoles, as pinpointed with arrows in the phalloidin staining (actin cytoskeleton) (Fig. 2G–J). Collectively these results show that both phage K1F-GFP and *E. coli* EV36-RFP bacteria can invade T24 human urinary bladder epithelial cells.

### Engineered phage K1F-GFP efficiently kills intracellular *E. coli* EV36-RFP bacteria in T24 human urinary bladder epithelial cells

**Figure 3:**
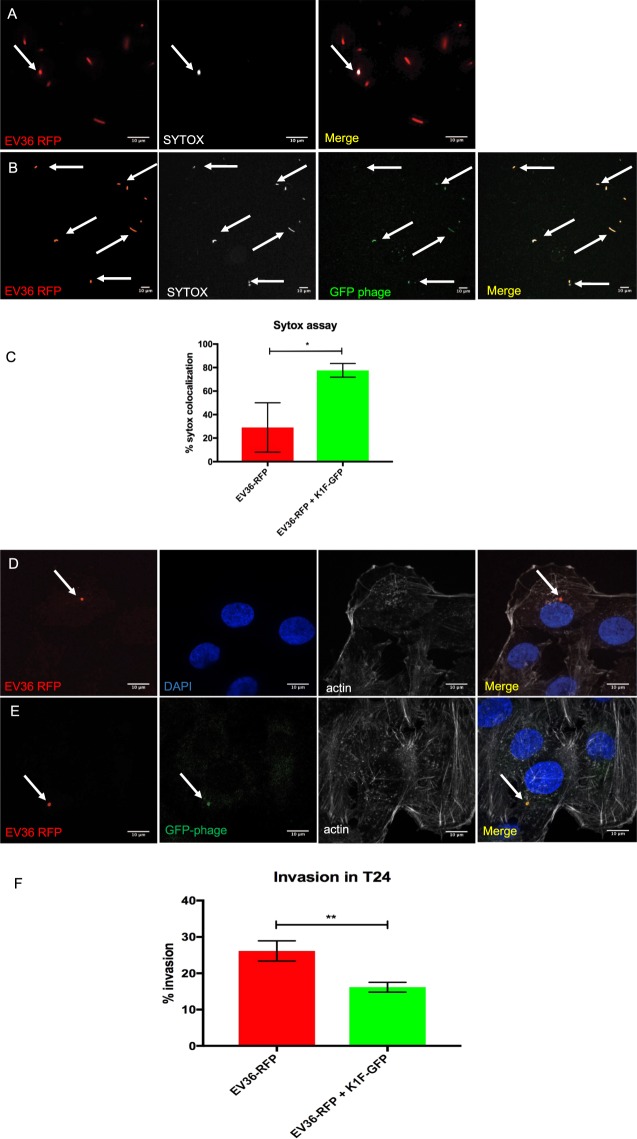
Phage K1F targets extracellular and intracellular bacteria in epithelial human cells. (**A,B**) Live confocal microscopy imaging of *E. coli* EV36-RFP bacteria stained with SYTOX Red Dead Cell stain (here shown in grey). Arrows refer to SYTOX stained cells. (**A**) Negative control sample containing *E. coli* EV36-RFP without phage K1F. (**B**) Sample containing *E. coli* EV36-RFP infected with fluorescent phage K1F*g10b::gfp*. (**C**) Quantification of SYTOX stained cells from live confocal imaging showing the percentage of *E. coli* EV36-RFP cells stained with SYTOX prior (29.1%) or upon fluorescent phage K1F addition (77.6%). Comparisons of means were carried out using a Student’s t-test with error bars representing +/− SD. Calculated probability values are p = 0.018 (*), n = 3. (**D–F**) Confocal imaging and quantification of intracellular *E. coli* EV36-RFP and fluorescent phage K1F in human epithelial cells. T24 cells have been stained with Phalloidin as a marker for F- actin and DAPI for DNA-rich nucleus. Each panel represents one channel of a single image. **‘**Merge’ panel shows all channels merged into one image. Each set of panels come from a single image. (**D**) T24 cells have been infected with *E. coli* EV36-RFP and further incubated with gentamycin to ensure the clearance of extracellular bacteria. The arrow indicates intracellular *E. coli* EV36-RFP bacteria. (**E**) T24 cells have been infected with *E. coli* EV36-RFP and fluorescent phage K1F*g10b::gfp* has been added. The arrows indicate intracellular *E. coli* EV36-RFP and intracellular fluorescent phage K1F co-localising. (**F**) Quantification of invasion experiments, showing that 26.1% (SD = 2.8) of T24 cells were invaded by intracellular bacteria and above half of those bacteria in total (16.2%, SD = 1.4) were targeted by fluorescent phage K1F*g10b::gfp*. Error bars indicate Standard Deviation. Comparisons of means were carried out using a Student’s t-test with error bars representing +/− SD. Calculated probability values are p ≤ 0.01 (**), n = 3.

Next, we applied SYTOX Red Dead Cell staining to investigate the ability of phage K1F-GFP to efficiently target and kill both intracellular and extracellular *E. coli* EV36-RFP bacteria. We found that upon phage addition, the percentage of intracellular dead bacteria (Fig. 3A,C) increases significantly (Fig. 3B,C). *E. coli* EV36-RFP showed 29.1% co-localisation with SYTOX (n = 3 experiments), whereas upon phage K1F addition, this was increased to 77.6% co-localisation with SYTOX (n = 3 experiments). Additionally, we observed co-localisation between bacteria (red), phage (green) and SYTOX (grey), indicating that the bacteria infected by bacteriophage were dying. These results were further confirmed with live time-lapse microscopy (Suppl. Fig. S8A,B). T24 cells were infected with *E. coli* EV36-RFP (Suppl. Fig. S8A) and in the absence of phage K1F-GFP, the bacteria continued to grow and damaged the human cells. In the presence of phage K1F-GFP (Suppl. Fig. S8B), the *E. coli* EV36-RFP infection cleared over time and dead *E. coli* EV36-RFP began to float in the medium and made aggregates that moved via Brownian motions. In order to further confirm that the reduction in the intracellular bacteria is a consequence of phage infection, we performed again the SYTOX assay, this time applying phage K1F on *E. coli* MG1655 bacteria (n = 3 experiments), which as shown previously are not sensitive to phage K1F (Suppl. Fig. S8B) and phage T7 on *E. coli* EV36 bacteria (n = 3 experiments), which as shown previously are not sensitive to phage T7 (Suppl. Fig. S1A). Our results (Suppl. Fig. S3), present that upon phage addition, in both cases, there was no statistical significant increase in the percentage of intracellular dead bacteria, measured by co-localisation with SYTOX. These results enhance our observations that the increase in intracellular *E. coli* EV36 dead bacteria upon K1F phage addition is due to phage specificity to its host and phage infectivity rather than degradation of the bacteria by the T24 epithelial human cells. We next incubated the culture with gentamycin^21,24^ in order to clear it from extracellular bacteria and subsequently quantified the amount of intracellular *E. coli* EV36-RFP in T24 cells (Fig. 3D,F) to be 26.1% (n = 3 experiments). Upon bacteriophage addition, it was observed that phage K1F-GFP could co-localise with intracellular *E. coli* EV36-RFP (Fig. 3E,F) and could target more than half (16.2%, n = 3 experiments) of the intracellular *E. coli* EV36-RFP (of 26.1% as above). These results show that engineered phage K1F-GFP can kill intracellular *E. coli* EV36-RFP in T24 human urinary bladder epithelial cells. To estimate the concentration of bacteria to be added to T24 epithelial cells and further understand the correlation between concentration of bacteria added for incubation to human cells and the fraction of cells containing intracellular bacteria, we incubated T24 human cells as previously with *E. coli* EV36-RFP at concentrations ranging from 5 × 10^6^ to 2 × 10^7^ CFU (Suppl. Fig. S4). Based on our observations upon quantification, the concentration of added bacteria strongly correlates with the fraction of human cells harbouring intracellular bacteria. More specifically, when incubated with *E. coli* EV36-RFP (5.65 × 10^6^ CFU/ml), 8.7% (n = 3 experiments) of T24 epithelial human cells were invaded, when incubated with *E. coli* EV36-RFP (1.13 × 10^7^ CFU/ml), 12.74% (n = 3 experiments) of human cells were invaded, whereas, when incubated with *E. coli* EV36-RFP (2.26 × 10^7^ CFU/ml), 22.74% (n = 3 experiments) of T24 epithelial human cells were invaded. Most of the invaded human cells (>95%) carried only a single bacterial cell within. The experiment was performed in biological triplicates and quantified by manually counting a minimum of 125 cells of each experimental condition. For the microscopy observation purposes, it was decided that bacterial concentration higher to 2.26 × 10^7^ CFU/ml on our experimental setting was not preferred, due to defects caused to human cells soon after incubation, and thus causing difficulties to properly quantify the internalised bacteria in single cells. Equally, bacterial concentrations lower to 5.65 × 10^6^ CFU/ml, resulted to a very small amount of intracellular bacteria and thus were not preferred either due to difficulties to identify intracellular bacteria in our microscopic observations.

To assess the dependence of the fraction of phage-containing human cells on the phage concentration, T24 cells were incubated with phage K1F-GFP, with PFUs ranging from approximately 1 × 10^5^ to 1 × 10^7^ (1.4 × 10^5^, 1.28 × 10^6^ and 1.27 × 10^7^). The samples were observed on the confocal microscope and the number of human cells containing phage-vacuoles were quantified and were further divided by the number of total cells imaged. Upon confocal observation and quantification of internalized phages K1F-GFP, it was estimated that final PFU of approximately 1.28 × 10^6^ resulted in 13.8% (n = 3 experiments) of phage K1F-GFP internalized in T24 human cells. PFU approx. 1 × 10^5^ gave weak GFP signal, which we had difficulty to quantify and at PFU 1.27 × 10^7^, we did not observe any difference compared to PFU 1.28 × 10^6^, apart from the fact that the GFP signal looked brighter, possibly indicating a higher number of intracellular phages/higher number of phage virions in these cells.

We therefore concluded that the concentration of phages added to the cell culture did not influence the fraction of human cells that have up-taken them, but most probably affected the number of phages per human cell.

Based on previous observations, showing that *C. difficile* bacteriophages are more virulent to their host in the presence of human cells^30^, we performed similar experiments in our system to investigate whether this is also the case with phage K1F and *E. coli* EV36 in the presence of T24 epithelial human cells. Indeed, it was observed (Suppl. Fig. S5A), that the concentration of planktonic bacteria upon phage K1F addition and in the presence of T24 human epithelial cells decreased more rapidly compared to the same conditions in the absence of human cells (at 90 min, 30 min after phage K1F addition, the concentration of planktonic bacteria in the presence of human cells dropped to 1.09 × 10^8^ CFU/ml (SD = 7.22 × 10^5^) compared to 3.13 × 10^8^ CFU/ml (SD = 2.16 × 10^7^) in the absence of human cells, with a statistical significance of p = 0.00008). Additionally, the PFU of phage K1F was higher in the presence of human cells when this was compared to the PFU of phage K1F in the absence of human cells (at 120 min, the PFU/ml of phage K1F in the presence of human cells was 5.13 × 10^11^ (SD = 1.46 × 10^11^), whereas in the absence of human cells was estimated 5.65 × 10^9^ (SD = 7.78 × 10^8^), with a statistical significance of p = 0.018) (Suppl. Fig. S5B). Interestingly, even when phage K1F was not added, the concentration of planktonic *E. coli* EV36 in the presence of human cells was shown to drop initially (at 90 min, the concentration of planktonic bacteria in the presence of human cells dropped to 6.21 × 10^8^ CFU/ml (SD = 5.91 × 10^7^) compared to 2.92 × 10^9^ CFU/ml (SD = 7.32 × 10^8^) in the absence of human cells (Suppl. Fig. S5A) possibly due to the process of invasion to the T24 epithelial human cells. Overall, these results confirm the previous observation^30^ that bacteriophages become more efficient in killing their host, in a human cell environment.

### Engineered phage K1F-GFP and *E. coli* EV36-RFP bacteria enter T24 human urinary bladder epithelial cells via phagocytosis and are degraded via LC3-associated phagocytosis

**Figure 4:**
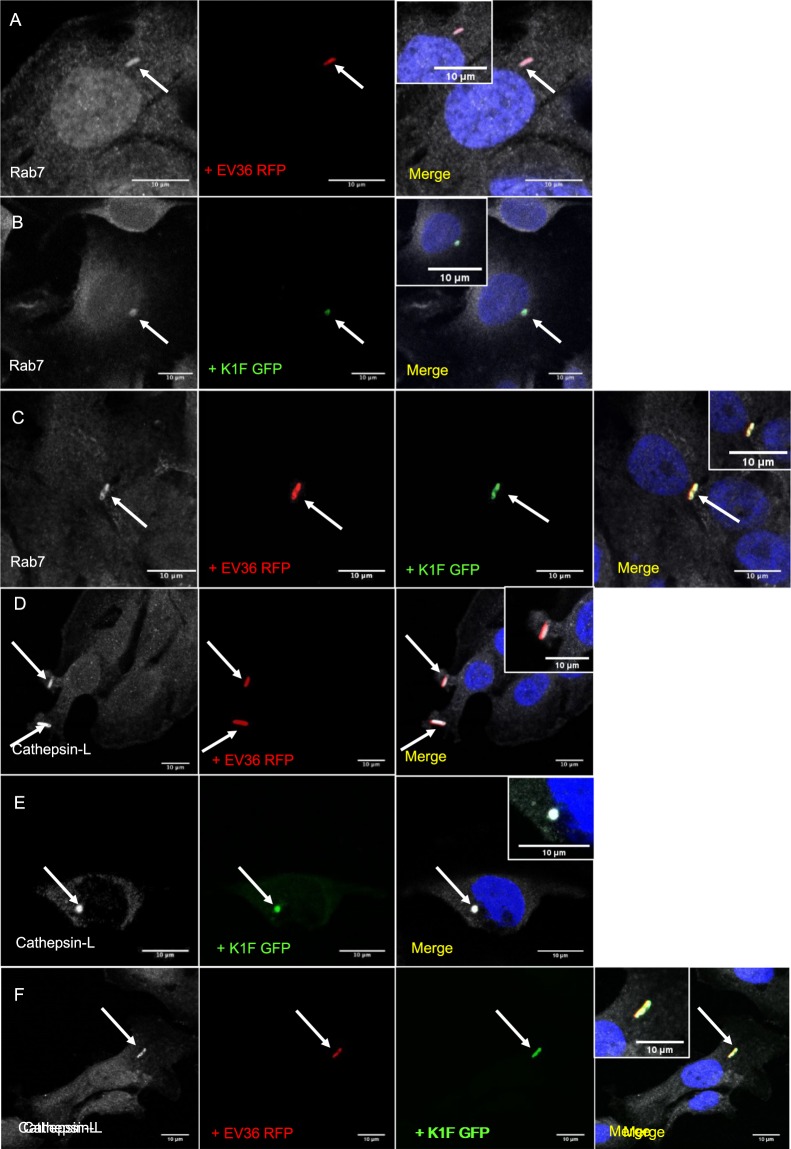
*E. coli* EV36 and phage K1F enter epithelial human cells via phagocytosis and are directed to lysosomes. (**A–C**) T24 cells infected with *E. coli* EV36-RFP (**A**), fluorescent phage K1F (**B**) or both (**C**), were fixed and stained with anti-RAB7 antibody, a marker for phagosomes. DAPI stain is shown in blue and anti-RAB7 antibody in grey. (**D–F**) T24 cells infected with *E. coli* EV36 RFP (**D**), fluorescent phage K1F (**E**) or both (**F**), were fixed and stained with anti-Cathepsin-L antibody, a marker for lysosomes. DAPI stain is shown in blue and anti-Cathepsin-L antibody in grey. Arrows represent the RFP-expressing bacteria, GFP-labelled phage and their co-localisation with the corresponding antibody.

Our results obtained up to this point indicated that both bacteria and bacteriophage enter human cells via their engulfment into vacuoles (Fig. 2). To examine this further, we incubated T24 human urinary bladder epithelial cells with either *E. coli* EV36-RFP bacteria (approximately 2 × 10^7^ CFU/ml), phage K1F-GFP (approximately 1 × 10^8^ PFU/ml) or both. The treated cells were subsequently fixed and stained with phagosomal markers, starting with an Rab7 antibody, which has been shown to localise on phagosomes and to be required for phagosomal maturation^31^. We showed that Rab7 co-localises with *E. coli* EV36-RFP (Fig. 4A), phage K1F-GFP (Fig. 4B) and both *E. coli* EV36-RFP and phage K1F-GFP in combination (Fig. 4C), suggesting that *E. coli* EV36-RFP and phage K1F-GFP enter T24 cells via phagocytosis.

**Figure 5:**
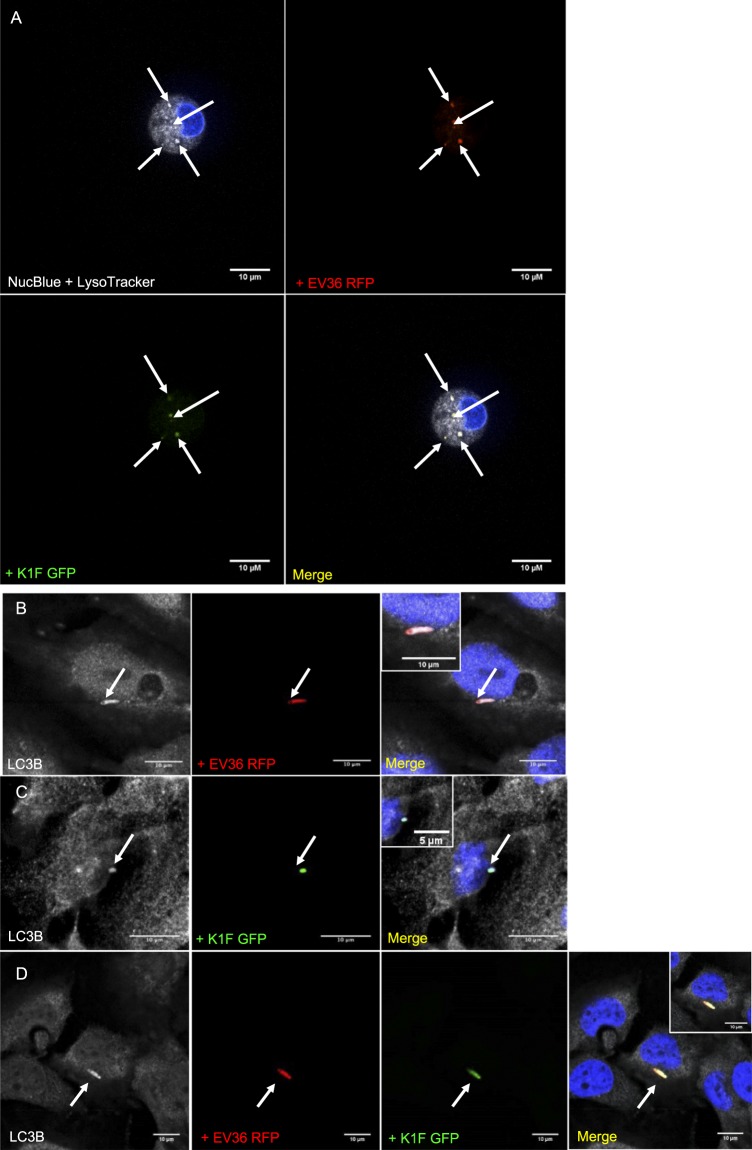
Degradation of *E. coli* EV36 and phage K1F via LC3-assisted phagocytosis. (**A**) Z-stack of live T24 cells infected with *E. coli* EV36-RFP bacteria and fluorescent phage K1F, stained with NucBlue Live ReadyProbes Reagent for the nucleus and Lysotracker Deep Red stain, a marker of acidic organelles in live cells. Shown here is a single image taken from a z-stack. (**B–D**) T24 cells infected with *E. coli* EV36-RFP (**B**), fluorescent phage K1F (**C**) or both (**D**), were fixed and stained with anti-LC3B antibody, a marker for LC3-assisted phagocytosis. DAPI stain is shown in blue and anti-LC3B antibody in grey. Arrows represent the RFP-expressing bacteria, GFP-labelled phage and their co-localisation with the corresponding stain/antibody.

It is known that phagosomes are fused with lysosomes where their content is degraded^32^. To this end, we next tested lysosomal markers to identify whether these co-localise with bacteria and bacteriophage that were previously phagocytosed. Using the same conditions as previously, treated T24 cells were stained with a Cathepsin-L antibody, a marker for lysosomes^33^. We observed that Cathepsin-L co-localised with *E. coli* EV36-RFP (Fig. 4D), phage K1F-GFP (Fig. 4E) and both *E. coli* EV36-RFP and phage K1F-GFP in combination (Fig. 4F), suggesting that both bacteria and bacteriophage are delivered upon phagosome maturation into lysosomes to be degraded. Additionally, live imaging of T24 cells stained with Lysotracker Deep Red stain, was performed to track lysosomes based on their acidic properties. We observed that *E. coli* EV36-RFP, phage K1F-GFP and the two in combination co-localise with lysosomes (Fig. 5A).

It has been shown that cells use some components of the autophagy machinery to process extracellular cargo, in a process called LC3-associated phagocytosis^32,34^. To test this, we treated T24 cells as previously and stained with an LC3 antibody^35^. It was indeed confirmed that both *E. coli* EV36-RFP (Fig. 5B), phage K1F-GFP (Fig. 5C) and both *E. coli* EV36-RFP and phage K1F-GFP in combination (Fig. 5D), co-localise with LC3, suggesting that these are degraded via LC3-assisted phagocytosis.

### *E. coli* EV36-RFP bacteria are degraded by xenophagy

**Figure 6:**
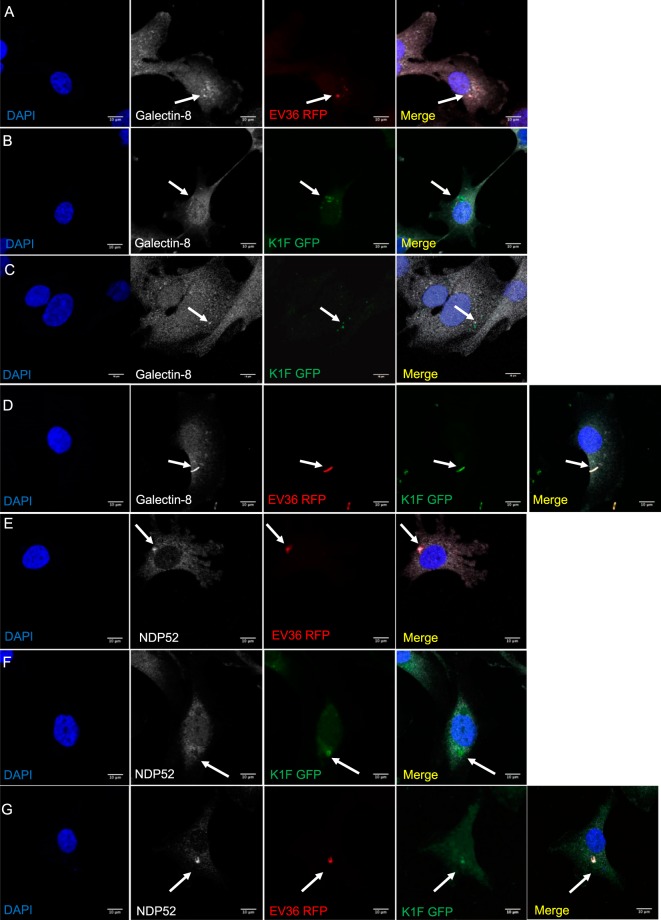
*E. coli* EV36 activates Galectin-8 dependent autophagy. (**A–C**) T24 cells were incubated with *E. coli* EV36-RFP alone (**A**), phage K1F-GFP alone (**B**), or *E. coli* EV36-RFP and subsequently with phage K1F-GFP (**C**). The cells were then fixed and stained with anti-Galectin-8/LGALS8 antibody as an autophagy marker. Arrows annotate *E. coli* EV36-RFP bacteria, fluorescent phage K1F and co-localisation with anti-Galectin-8 antibody. Images are representative of three independently performed experiments. **D–G**. T24 cells were incubated with *E. coli* EV36-RFP alone (**D**), phage K1F-GFP alone (**E,F**), or *E. coli* EV36-RFP and subsequently added phage K1F-GFP (**G**). The cells were then fixed and stained with anti-NDP52/CALCOCO2 antibody as an autophagy marker. Arrows annotate *E. coli* EV36-RFP bacteria, fluorescent phage K1F and co-localisation with anti-NDP52 antibody. Images are representative of three independently performed experiments.

It has been shown previously that intracellular bacteria can escape the initial phagosome and activate xenophagy (anti-bacterial autophagy) through selective autophagy receptors^32^. To test this, we incubated T24 human urinary bladder epithelial cells with either *E. coli* EV36-RFP bacteria (approximately 2 × 10^7^ CFU/ml), phage K1F-GFP (approximately 1 × 10^8^ PFU/ml) or both. The treated T24 cells were fixed and initially stained with a Galectin-8 antibody, which has been found to participate in xenophagy via the targeting of damaged vesicles upon bacterial invasion^36^. Here, Galectin-8 was found to co-localise with 50.6% n = 4 experiments) of intracellular *E. coli* EV36-RFP (Fig. 6A), but only with 24.4% (n = 4 experiments) of intracellular phage K1F-GFP (Fig. 6B,C). When intracellular *E. coli* EV36-RFP was infected with phage K1F-GFP, the co-localisation with Galectin-8 was increased to 100.0% (n = 3 experiments) (Fig. 6D).

We then repeated the same sets of experiments, this time staining with an NDP52^37^ antibody, a xenophagy receptor. Upon performing the experiment, we could see NDP52 co-localised with 47.8% (n = 3 experiments) of intracellular *E. coli* EV36-RFP (Fig. 6E), suggesting that cytosolic *E. coli* EV36-RFP are degraded via xenophagy. In cells incubated with phage K1F-GFP, it was observed that intracellular bacteriophages alone do not co-localise with NDP52 (n = 3 experiments) (Fig. 6F). In the presence of both bacteria and phages (Fig. 6G), the co-localisation of NDP52 with intracellular *E. coli* EV36-RFP infected with phage K1F-GFP, quantified from three different experiments, was found to be 24.9% (n = 3 experiments).

**Figure 7:**
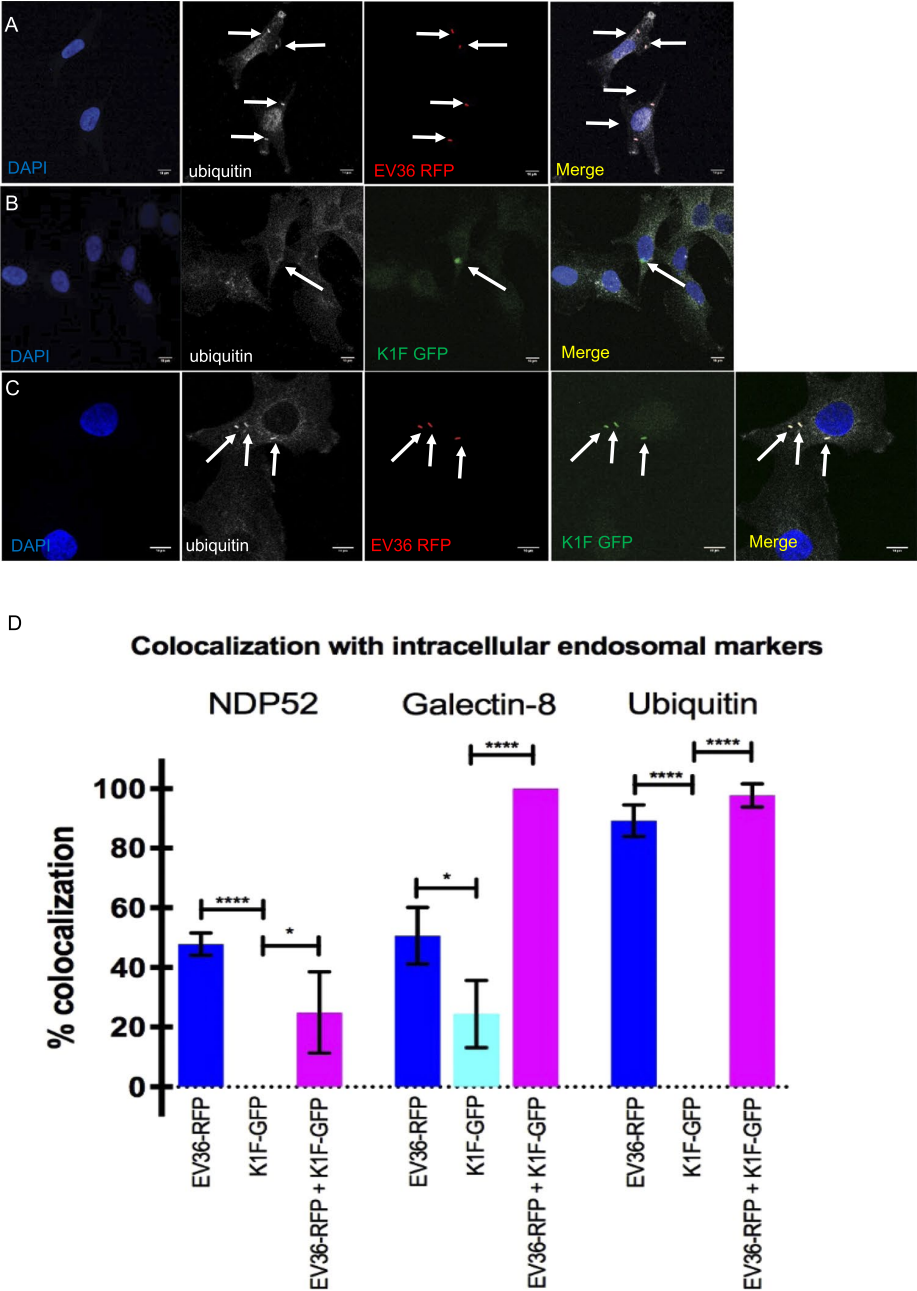
Phage K1F in the absence of *E. coli EV36* cannot activate autophagy. (**A–C**) T24 cells were incubated with *E. coli* EV36-RFP alone (**A**), phage K1F-GFP alone (**B**), or *E. coli* EV36-RFP and subsequently added phage K1F-GFP (**C**). The cells were then fixed and stained with anti-ubiquitin antibody as an autophagy marker. Arrows annotate *E. coli* EV36-RFP bacteria, fluorescent phage K1F and co-localisation with anti-ubiquitin antibody. Images are representative of three independently performed experiments. (**D**) Quantification of co-localisation of endosomal markers with *E. coli* EV36-RFP and phage K1F-GFP alone and in combination. A Student’s t-test corrected for multiple comparisons using the Holm-Sidak method was performed using GraphPad Prism 7. The calculated probability values (p-values) are displayed as p ≤ 0.05 (*), p ≤ 0.01 (**), p ≤ 0.001 (***), p ≤ 0.0001 (****) and not statistical significant p ≥ 0.05 (ns). n ≥ 3.

Finally, we repeated the same sets of experiments, this time staining with an ubiquitin antibody, as a marker for ubiquitinated proteins^34^. Ubiquitin was found to co-localise with 89.2% (n = 3 experiments) of intracellular *E. coli* EV36-RFP (Fig. 7A), but no co-localisation (n = 3 experiments) was observed with intracellular phage K1F-GFP (Fig. 7B). On the contrary, in the presence of both phage and bacteria (Fig. 7C) the co-localisation with ubiquitin increased to 97.8% (n = 3 experiments), indicating that the sole presence of bacteria increases ubiquitination dramatically.

These experiments were repeated in triplicates at minimum and the quantification corresponds to the average co-localisation with intracellular bacteria or bacteriophage across all experiments (Fig. 7D). Negative controls for all the secondary antibodies used for the co-localisation assays, verify the validity of these results (Suppl. Fig. S6C,D and E). Based on the above results, we propose that both *E. coli* EV36-RFP and phage K1F-GFP invade T24 epithelial human cells via phagocytosis and they can be degraded via LC3-assisted phagocytosis. When *E. coli* EV36-RFP escape the phagosome and become cytosolic they can activate xenophagy, whereas the bacteriophage alone cannot activate xenophagy. In the presence of both phage and bacteria, xenophagy is activated.

## Discussion

Bacteriophages are the most abundant viruses residing in the human body even in the absence of disease^38–40^. In various instances, the presence of bacteriophages within the human body and especially in the gut^41–45^ and in the bladder^41^, is regarded to function as a protective mechanism to the human body in the control of pathogens^46,47^. Recent studies have shown that bacteriophages can even invade human cells, either randomly, via transcytosis across epithelial cell layers^7^, via direct cellular interactions the bacteriophages might have with mammalian cells^6,8,48,49^ or via phagocytosis by macrophages^50^. In this study, we have engineered a fluorescent phage K1F that specifically targets the K1 polysaccharide capsule of *E. coli* EV36. We inserted a *gfp* gene into the phage genome by combining *in vivo* homologous recombination with CRISPR/Cas-selection. The engineering of fluorescent bacteriophages with the insertions of a fluorescent gene in their genome has been applied before, using various methodologies and different bacteriophages, such as T7^51^, PP01^52^, T4^53^, P22^54,55^ as well as the mycobacteriophages D29^56^ and DS6A^57^, where the fluorescent protein is either expressed in the capsid of the phage or in a non-essential region of its genome. We report for the first time the engineering of a fluorescent phage K1F and we have applied for the first time CRISPR/Cas to select for fluorescent bacteriophages. Even though we initially tried to integrate the *gfp* gene into the *gene 10* (*g10*) of the phage’s genome, encoding for the major capsid protein of phage K1F, we were not able to isolate recombinant phage due to its fitness defect. This was confirmed by the observation of the formation of very small plaques obtained by plating the phage mix. Plaques with a ten-fold reduced diameter were found in frequencies of 1/94 and 1/135. PCR analysis of these plaques however yielded fragments of unexpected size, indicating the incorrect genomic integration of *gfp*. Our inability to fuse GFP to the C-terminal of gp29 may have resulted from at least two factors. First, the fusion of *gfp* to the 3′ of *gene10* interrupts the ORF of *gene10b*. The lack of isolated plaques harbouring the correct construct most likely indicates that infective virions cannot assemble without the minor capsid protein. The presence of both wild type and recombinant phage genomes in the same cell however may allow the packaging of the recombinant DNA into wild type heads, explaining the existence and PCR-positivity of the phage K1F + phage K1F*g10::gfp* mix. Second, the lack of a linker peptide between the capsid protein and GFP in the *g10::gfp* construct could severely reduce the fitness of the phage by inhibiting proper capsid protein folding^58^. To avoid these caveats, we attempted to fuse the *gfp* to gene 10b (*g10b*), resulting from a -1 translational frameshift in the 3′ region of *g10*, encoding for the minor capsid protein of the phage and the two remaining constructs were designed in a way to harbour a 3xGGGGS flexible linker connecting the fused proteins. For phage genome editing, we relied on the strategy of providing the donor DNA on a plasmid residing in the bacterial strain used to grow the targeted phage, combined with the CRISPR/Cas selection of recombinant phages^16,17^. With the above methodology, we managed to generate a stable recombinant fluorescent phage K1F. This has enabled us to observe the invasion of this phage into T24 human epithelial cells via phagocytosis, which was confirmed after our initial observations of invasion of fluorescent phage K1F into vacuoles, with the co-localisation of phage K1F with anti-Rab7 antibody, a protein known to localise into phagosomes and to participate in phagosome maturation^31^. The same was observed also for the *E. coli* EV36-RFP strain, which was also shown to invade T24 epithelial cells via phagocytosis and co-localise with anti-Rab7. It has been shown previously that *E. coli* K1 invades epithelial cells in a process depending on cytoskeletal rearrangements^24^ and regulated by the expression of the K1 capsule^21^ or via transcytosis^59^, assisted by the outer membrane protein expressed in *E. coli* K1^60^. We clearly present here that the phage K1F by this invasion mechanism can efficiently kill the host bacteria in human cellular environment and importantly can also target intracellular *E. coli* EV36 bacteria. In order to further verify that the killing of the intracellular *E. coli* EV36 is attributed to the efficiency and specificity of phage K1F rather than the potential bacterial degradation by T24 epithelial cells, we performed control experiments with phage T7 and phage K1F coupled with bacterial strains non-sensitive to these phages (*E. coli* EV36 and *E. coli* MG1655 respectively) and upon these conditions we did not observe any increase in the killing of the bacterial strains upon addition of the aforementioned phages. These results confirmed that phage K1F is able to target intracellular host bacteria in human cell environment. This is a novel finding and very informative on the *in vitro* mechanism of phage therapy, providing knowledge that can accelerate this field in the future. Previous observations suggest that phage phiCDHS1 is more virulent to its host *C. difficile* bacteria in epithelial cell environment^30^. Our results enhance this finding, since we also observed that phage K1F presented higher killing efficiency towards its host *E. coli* EV36 bacteria in the presence of T24 epithelial human cells. Taken together, these results further confirm the hypothesis that there are specific interactions between phages and humans cells, possibly in forms of recognition receptors present in phages and human cells, which facilitate the bacterial clearance further during phage treatment^30^.

Lysosomes (phagolysosomes) are considered the endpoint in the phagocytosis where the mature phagosome fuses with a lysosome and any particles that are contained inside are completely degraded. We showed that both the phage and the bacteria upon phagocytosis, co-localise with Cathepsin-L, an endopeptidase enzyme that plays an important role in lysosome function^33^ and this was further confirmed with their co-localisation with Lysotracker Deep Red stain, which also a marker for lysosomes. This is indicative that phage K1F and *E. coli* EV36 bacteria enter the lysosomal degradative pathway, possibly as the result of constitutive maturation of phagosomes. In all the above cases, we showed that bacteria and phage together and independently were found in phagosomes and lysosomes. Uropathogenic *E. coli* bacteria have been shown to subvert the human innate immune system by escaping the phagosome^61^. When this vacuole is damaged by effectors released by the bacterium the glycan molecules that are typically enclosed within the cell compartment are exposed to the cytosol. The cell combats this via antibacterial autophagy (xenophagy) mechanisms^32^. This is typically via galectin-8 or ubiquitin dependent autophagy which both recruit the microtubule-associated protein 1A/1B-light chain (LC3). Our results suggests that *E. coli* EV36 bacteria and phage K1F are able to escape the phagosome and are targeted by autophagy mechanisms. On the contrary, in the presence of phage K1F alone, co-localisation was observed only with Galectin-8 in approximately 20% of the internalized phage, whereas 50% of bacteria alone were shown to co-localise with Galectin-8 and in the presence of both bacteria and phage this was increased to 100%. Phage K1F alone was not shown to co-localise at all neither with ubiquitin nor NDP52, both further down on the xenophagy pathway, indicating that bacteriophage alone cannot activate autophagy^32^. From these results, it seems that bacteriophage is degraded via LC3-assisted phagocytosis, rather than autophagy. This would suggest that the bacteriophage does not have the necessary machinery to escape the phagosome in which it was contained. Bacteriophages are found not to be toxic to human cells^30^ and thus we hypothesize based on our results that unlike their bacterial hosts, bacteriophages are not considered to be harmful for the human body. This hypothesis could explain the differential activation of mechanisms of degradation between phages and bacteria. The host pathogenic bacteria are known to be harmful for the human body and thus activate autophagy^32^. To our knowledge, there are no reports to this date on the mechanisms that bacteriophages are degraded by. This is the first report presenting a mechanism of bacteriophages’ degradation.

Our results overall reveal for the first time the mechanism via which bacteriophages that invade T24 human cells get degraded in comparison to the mechanism of invasion and degradation of their host bacteria. The fluorescent phage K1F that we have engineered provides an excellent tool to discover the pathway that bacteriophages follow in their interaction with bacteria and human cells. Finally, the engineering of this human tissue model system of phage therapy in *E. coli* EV36, mimicking the *E. coli* O18:K1:H7^29^, a well-defined bacterial strain accountable for a multitude of conditions, has enabled us to unfold the cellular processes underpinning phage therapy. Our study provides an essential platform for engineering synthetic phages against *E. coli* or other antibiotic resistant bacteria as an innovative tool in the AMR fight.

## Methods

### Tissue cell culture

The human urinary bladder epithelial cell line, T24 (HTB-4), was acquired from LGC Standards (UK) an ATCC (American Type Culture Collection) affiliate. This cell line was originally derived from a bladder transitional cell carcinoma of a female patient.

The T24 cells were cultured in uncoated T75 flasks containing McCoy’s 5A (Modified) Medium (Gibco, MA) supplemented with 10% v/v Foetal Bovine Serum (FBS) (Labtech International, UK) and 1% v/v Penicillin-Streptomycin and were maintained at 37 °C in 5% CO_2_ under a humidified atmosphere.

### Purification of bacteriophages

A single-clone preparation of phage K1F (kindly provided by Dr Dean Scholl^27^) was obtained by three consecutive infections of *E. coli* EV36 which is a K12/K1 hybrid developed by conjugation of Hfr kps + strain^29^ (kindly provided by Dr Eric R. Vimr). *E. coli* EV36 was used as an *E. coli* O18:K1:H17 analogue host to grow and purify phage K1F from a single plaque.

The single-clone phage K1F preparation was propagated to a high concentration over many infection cycles of cultures with increasing *E. coli* EV36 concentration. Each propagation culture was centrifuged at 3220 g for 15 minutes at 4 °C after bacterial clearance. The final culture was incubated with 0.2 M NaCl on ice for 1 hour to release phage particles from bacterial membranes and centrifuged at 5000 g for 45 minutes at 4 °C. The supernatant was recovered and incubated with 10% w/v PEG8000 for 16–18 hours at 4 °C to precipitate phage particles. The PEG solution was centrifuged for 25,000 g for 1 hour at 4 °C and the pellet was resuspended in a small volume SM-buffer^62^.

A CsCl density gradient was prepared in water with three solutions in densities of 1.7, 1.5 and 1.3 g/ml. CsCl was added to phage solution to achieve a density of 1.3 g/ml. The CsCl solutions were added to the centrifuge tube in equal volumes starting with the heaviest and subsequently were centrifuged at 125,000 g for 20 hours at 4 °C.

The resulting blue/grey band was extracted and transferred to a clamped dialysis tube with MWCO of 14 kDa and dialysed for 16–18 hours in a SM-buffer with high concentration of NaCl (1 M) followed by 2 times 2 hours in a SM-buffer with low concentration of NaCl (100 mM). Following dialysis the purified bacteriophage was stored at −20 °C.

### Determination of phage infec**t**ion efficiency in liquid culture

The phage infection efficiency and host selectivity were determined in a liquid culture assay. Bacterial cultures of *E. coli* EV36 strain and *E. coli* K-12 MG1655 (ATCC-47076) wild-type^63^, were inoculated in lysogeny broth (LB)^64^ and incubated in a rotating incubator at 37 °C.

The optical density was measured at 600 nm in 30 minute intervals. Cultures were inoculated with 1 µl wild type phage K1F (PFU 10^8^) or 1 µl T7^65^ (PFU 10^10^) phages once they reached their exponential phase after approximately two hours.

### Transmission electron microscopy (TEM)

400 mesh copper grids with carbon graphite coating were cleaned and hydrophilized by glow discharge. A 10 µl drop of purified phage K1F was applied to the centre of the mesh and was incubated for 1 minute. The sample was removed and the mesh washed twice with 10 µl drops of water and finally negatively stained with 10 µl 2% uranyl acetate for 1 minute. Images were acquired using the Jeol 2100 transmission electron microscope.

### Construction of *E. coli* EV36-RFP bacteria

In order to visualise the location and concentration of bacteria in a human cell environment, fluorescent *E. coli* EV36-RFP bacteria were constructed. *E. coli* EV36 bacteria were made electrocompetent as shown previously^66^ and were transformed with plasmid pSB1C3 (Registry of Standard Biological Parts. http://parts.igem.org/Main_Page) expressing the mRFP1 protein. Cells containing RFP were selected for with chloramphenicol (Cm: 25 µg/ml) and IPTG induction (0.5 mM).

### Construction of genetically modified fluorescent bacteriophages

#### Strains, buffers and media

Phage dilutions were made in buffer Φ80 + containing 0.1 M NaCl, 0.01 M Tris (pH 7.9), 0.01 M CaCl_2_ and 0.01 M MgCl_2_^67^. TBE buffer contained 45 mM Tris, 45 mM boric acid and 1 mM EDTA^64^. Bacteria were grown in lysogeny broth (LB)^64^. To make soft agarose for phage titering and plaque assays, LB-agar plates were overlain with 0.5% Seakem LE agarose (Lonza, Basel, Switzerland) containing 5 mM CaCl_2_ and 5 mM MgCl_2_. Antibiotics (Sigma-Aldrich, St. Louis, MO, USA) were used in the following concentrations: ampicillin (Ap): 50 µg/ml, chloramphenicol (Cm): 25 µg/ml.

#### Plasmid construction

Plasmid pSB6A1 was obtained from Registry of Standard Biological Parts. (http://parts.igem.org/Main_Page). Plasmid pCas9 (Addgene #42876) was the kind gift of Luciano Marraffini (The Rockefeller University), obtained via Addgene. The three donor plasmids used for phage K1F genome editing, pSBGFP, pSBN and pSBC3 were constructed by digesting a synthetic gene cassette ordered from Integrated DNA Technologies (Leuven, Belgium) with EcoRI and PstI and ligating to the corresponding restriction sites of the pSB6A1 plasmid. The synthetic gene cassettes comprised the superfolder Green Fluorescent Protein (GFP) gene flanked by two 150 bp-long homology boxes corresponding to segments of the phage K1F genome in the gene 10 region (for exact DNA sequences, see Table S1).

The three selection plasmids, pCas9g10, pCas9K1FC1 and pCas9K1FC2 were constructed by ligating the respective CRISPR spacers into the BsaI site of pCas9, as described previously^68^. The CRISPR spacers were designed by manually searching for 30 nt long stretches of the wild type phage K1F genome that span the integration site of GFP and end with an NGG. The spacers were synthesized as pairs of complementary oligonucleotides carrying the ends required for cloning (Table S2).

#### Phage propagation and titering

Routine bacteriophage procedures were conducted as described previously^69^. Briefly, for phage propagation, *E. coli* EV36 cells were grown by agitating at 37 °C in LB medium supplemented with 5 mM CaCl_2_ and 5 mM MgCl_2_ and were mixed with 10^6^ plaque forming units (PFU) of phage K1F upon reaching an OD_600_ value of 0.5. Complete bacterial lysis was usually observed within 1–2 h of further shaking. At this point, 50 µl of chloroform was added and the lysate was shaken for 5 min, followed by pelleting of the cell debris and filtration of the supernatant with a 0.22 um Millex GP PES filter (Merck-Millipore, Carrigtwohill, Ireland) to obtain a cell-free phage lysate.

For phage titering and plaque assays, LB plates were overlain with 3 ml of molten soft agarose (cooled to 42 °C) containing 100 µl of midlog-phase *E. coli* EV36 cells. After solidification, 10 µl samples from each member of a dilution series of the phage lysate, ranging from the concentrated solution to 10^−9^ dilution was pipetted on top and let to dry. Individual plaques were already visible within the *E. coli* lawn at the appropriate dilutions after 4 h of incubation at 37 °C. The appropriately diluted phage lysate was then mixed with 100 µl of midlog-phase *E. coli* EV36 cells, mixed with 3 ml of molten soft agarose (cooled to 42 °C) and immediately poured onto LB plates at room temperature. After solidification, plates were incubated overnight at 37 °C. The plaque numbers counted the next day were used to calculate phage titers in PFU/ml. To estimate the fraction of recombinant phages in a phage mix, PCRs using primers GFPfw and g11rev were carried out on 1 µl samples from the dilution series of the phage mix. The ratio of the highest dilution factor still yielding a positive PCR and the phage titer (expressed as PFU/µl) gave the approximate fraction of recombinants within the phage mix.

#### Phage genome engineering via in vivo homologous recombination

To integrate the GFP gene into the genome of phage K1F, wild-type (wt) phages were propagated on *E. coli* EV36 cells harboring the appropriate donor plasmid for 1–3 rounds, as described above. The presence of recombinant phages in the obtained mixed lysate was verified by PCR analysis of the 1000-fold diluted lysate using primers GFPfw and g11rev (Table S2). To acquire pure recombinant phage cultures, the mixed lysate was propagated on *E. coli* EV36 carrying the appropriate selection plasmid. The obtained phage lysate was titered and the appropriate dilution was plated on *E. coli* EV36 to obtain individual plaques. In a plaque-assay, 10–20 plaques were aspirated with cut-off pipette tips as agarose plugs and released each into 40 µl of TBE buffer. After 4 h of incubation at 42 °C and a short vortexing, 0.5 µl of each sample was used as a template in PCR reactions using primers GFPfw and g11rev. The phage suspensions yielding a correct PCR product were titered and plated again on *E. coli* EV36 for a second round of plaque assay to warrant their purity. Positive phage suspensions obtained this way were propagated on *E. coli* EV36 to generate recombinant phage stocks to be further used in infection assays analyzed by microscopy.

#### Sequencing of recombinant bacteriophages

The sequence of the engineered K1F*g10b::gfp* phage genome in the vicinity of the *gfp* insertion was verified by Sanger capillary sequencing using the ABI Big Dye Terminator v3.1 kit and a 3500 Series Genome Analyzer (Life Technologies-Thermo Fisher Scientific, Waltham, MA, USA). First, the region of interest was PCR-amplified using primers g9fw and g11rev. This 2591 bp-long PCR product was purified using the PCR Advanced PCR Cleanup System (Viogene, Taiwan, China) and sequenced with primers g9fw, g10fw, AS504 and g11rev. The obtained reads were aligned with the expected phage sequence using the Serial Cloner v2.6.1 (Serial Basics Inc.) to assemble to empirical *g10b::gfp* sequence map. The empirical sequence matched the expected sequence except for a C > T transition at 22264, resulting in a Thr377Ile mutation in the C terminal of *g10b*.

### Immunofluorescence and confocal microscopy

Fluorescent immunocytochemistry was performed to identify the association of bacteria and phage with biological markers of the endolysosomal and autophagy pathways.

For invasion assays, the T24 cells were seeded onto uncoated 22 × 22 mm coverslips in 6-well plates at a density of approximately 4 × 10^4^ cells/cm^2^ in McCoy’s 5A (Modified) Medium (supplemented with 10% v/v FBS only, without antibiotics at 37 °C) and were allowed to settle for 24 hours.

The culture media were aspirated and replaced with Leibovitz media (Lonza, Switzerland) that sustain cell viability in the absence of CO_2_ equilibrium and the cells were moved to an incubator appropriate for bacterial infections. While maintaining the confluent cultures at 37 °C, the cultures were incubated with *E. coli* EV36-RFP at OD 600 nm of approximately 0.4 to 0.6, which were added to 1 ml of Leibovitz media^21,24^ for 60 minutes following incubation with phages at PFU 1 × 10^8^ for 60 minutes. In parallel, control cultures were incubated with bacteria or phage alone for 60 minutes. For the assays estimating the number of intracellular bacteria, upon infection, the cells were washed with PBS and fresh medium containing 100 µg/ml gentamycin was added for further 2 hours to kill the extracellular bacteria, prior to bacteriophage addition^21,24^. Phage K1F-GFP was then added as previously for 60 minutes. Immediately thereafter, the cultures were fixed in 4% paraformaldehyde (PFA) (ThermoFisher Scientific, MA), permeabilised in ice-cold PEM buffer with 0.05% Saponin and quenched with 50 mM NH_4_Cl in PBS. Appropriate wash step with PBS was performed between each step^70^. In order to estimate further the correlation between the number of intracellular bacteria and the concentration of bacteria added in human cells, T24 human cells were incubated with *E. coli* EV36-RFP at approximate concentrations ranging from 5 × 10^6^ to 2 × 10^7^ CFU/ml for 2 hours. The cells were fixed and further treated as described above.

The fixed cells were stained with the following primary antibodies diluted in 0.05% Saponin in PBS for 45 minutes at room temperature; 40 µg/ml Anti-RAB7 (Bioss Inc, MA), 1 µg/ml Anti-Cathepsin L (Abcam, UK), 5 µg/ml Anti-LC3B (Sigma-Aldrich, MO), 1 µg/ml Anti-CALCOCO2/NDP52 (Abcam, UK), 1 µg/ml Anti-Galectin-8 (R&D Systems, MN) or 1 µg/ml Anti-Mono- and polyubiquitinylated conjugates monoclonal antibody (FK2) (Enzo Life Sciences).

This was followed by conjugation with secondary Cy5 Affinipure Donkey Anti-Goat, Anti-Rabbit or Anti-mouse (Jackson ImmunoResearch, PA). Finally, the stained cells were mounted on microscope slides using Flouroshield Mounting Medium (Abcam, UK) containing DAPI nuclear stain.

Cultures containing GFP tagged bacteriophages were further enhanced with a GFP-Booster (Chromotek, Germany) at a concentration of 5 µg/ml alongside with the conjugation with secondary antibodies. In the context of visualising bacteria and phage in the cell environment without endolysosomal markers, an actin filament stain, Phalloidin CF680R Conjugate (Biotium, CA), was used at a concentration of 5 µg/ml.

All fixed cells were imaged using the Zeiss LSM 880 confocal microscope with Airyscan, with fluorophore excitation at the following wavelengths: DAPI at 405 nm, GFP at 488 nm, RFP at 561 nm and far-red (Cy-5) at 633 nm.

Quantification was performed by manually counting 10–20 images of each experimental condition. A minimum of 200 cells were counted of each experimental condition.

### Live cell imaging

The T24 cells were seeded onto uncoated 35 mm glass bottom microscope dishes (ThermoFisher Scientific, MA) at a density of approximately 4 × 10^4^ cells/cm^2^ in McCoy’s 5A (Modified) Medium (supplemented with 10% v/v FBS only) and were allowed to settle for 24 hours.

The culture media were aspirated and replaced with Leibovitz medium (Lonza, Switzerland) that sustain cell viability in the absence of CO_2_ equilibrium. While maintaining the confluent cultures at 37 °C, the cultures were incubated with *E. coli* EV36-RFP bacteria and phage K1F-GFP as previously. For the SYTOX Bacterial Death Assay, *E. coli* EV36-RFP at OD 600 nm of approximately 0.4 to 0.6, were added to 1 ml of Leibovitz media for 60 minutes. Fluorescent phage K1F was added, at PFU 1 × 10^8^_,_ for another 60 minutes. SYTOX Red dead cell stain (final conc. 5 nM) and 2 drops of NucBlue Live (ThermoFisher Scientific, MA) per 1 ml of Leibovitz medium was added to both samples for the last 20 minutes of the incubation. After the 20 minutes of SYTOX incubation, the cells were ready to be visualized under the Andor/Nikon Spinning Disk Confocal Laser Microscope.

Quantification was performed by manually counting 30–50 images of each experimental condition. A minimum of 200 cells were counted of each experimental condition.

For the live cell imaging with SiR tubulin (SPIROCHROME, Cytoskeleton, Inc.), T24 cells were seeded as previously onto uncoated 35 mm glass bottom microscope dishes (ThermoFisher Scientific, MA). 1 µL of SiR-tubulin and 1 µL Verapamil were added to 1 ml of McCoy’s 5A (Modified) Medium (supplemented with 10% v/v FBS only, free from Pen/Strep). This was added to the glass dishes containing confluent T24 cells to stain them for tubulin. After 1-hour of incubation the media were aspirated off to decrease the background of the SiR-tubulin staining and were infected with *E. coli* EV36-RFP bacteria and/or phage K1F-GFP as previously. In the last 20 minutes of infection, NucBlue Live (ThermoFisher Scientific, MA) was added as previously to stain for the nucleus. The samples were then visualized under the Andor/Nikon Spinning Disk Confocal Laser Microscope. In the live cell imaging provided in Supplementary Figures/Videos 7 and 8, when timing was different, it is mentioned on the corresponding figure legends.

### ***E. coli*** EV36-RFP and phage K1F-GFP concentration dependent infection in T24 cells

T24 cells were seeded on uncoated coverslips in 6-well plates as described previously.

T24 cells maintained in Leibovitz media at 37 °C were incubated with *E. coli* EV36-RFP at approximate concentrations ranging from 5 × 10^6^ to 2 × 10^7^ CFU/ml and incubated for 2 hours. The cells were gently washed twice in PBS to remove planktonic bacteria and were subsequently fixed and permeabilised and stained with Phalloidin CF680R Conjugate as described in immunofluorescence and confocal microscopy section. The same process was repeated with phage K1F-GFP: 1 µl, 10 µl and 100 µl of phage K1F-GFP (stock PFU 1.4 × 10^8^) was added in wells containing T24 cells (corresponding final PFUs/ml: 1.4 × 10^5^, 1.28 × 10^6^ and 1.27 × 10^7^) and incubated for 1 h. The cells were then treated as previously and were consequently observed with confocal microscopy.

### Time course measurements of planktonic bacteria and free phages

T24 cells were seeded in 6-well plates at a density of approximately 2.2 × 10^4^ cells/cm^2^ in McCoy’s 5A (Modified) Medium supplemented with 10% v/v FBS and were allowed 48 hours to settle. The culture media was aspirated and replaced with Leibovitz media and maintained at 37 °C before commencing the experiment. *E. coli* EV36 and phage K1F were added to corresponding wells at approximate final concentrations of 5 × 10^6^ CFU/ml and 3 × 10^3^ PFU/ml respectively. Sampling was performed at 30 minutes intervals over a period of two hours.

Serial dilutions and plating for CFU determination was performed immediately after sampling, whereas samples taken for PFU determination were centrifuged at 3220 rpm for 15 minutes at 4 °C, immediately after sampling, to remove bacterial debris. The phage containing supernatant was stored at 4 °C for plating collectively, following the two-hour sampling period. CFU concentrations were determined by drop assay, plating 4 × 20 µl drops of each 10-fold dilution on LB plates and incubating these for 24 hours at 37 °C. Dilutions selected for CFU quantification accounted for a minimum of 80 colonies of a dilution. PFU concentrations were determined by overlay assay, plating 100 µl phage of each 10-fold dilution with 300 µl host log-phase bacteria in soft agar as described previously under phage propagation and titering. Dilutions selected for PFU quantification accounted for a minimum of 35 plaques of a dilution.

Each experiment was performed in biological triplicates and presented in graphs as the average +/− standard deviation of these.

### Statistical analysis

Values are shown as the mean +/− SD of a minimum of three independent experiments in all figures. In liquid infection assays (Suppl. Fig. S1A), comparisons of means were carried out using a student t-test on the SPSS platform. In imaging quantification (Fig. 3C,F, 7D and Suppl. Fig. S4), a Student’s t-test corrected for multiple comparisons using the Holm-Sidak method was performed using GraphPad Prism 7. The calculated probability values (p-values) are displayed as p ≤ 0.05 (*), p ≤ 0.01 (**), p ≤ 0.001 (***), p ≤ 0.0001 (****) and not statistical significant p ≥ 0.05 (ns).

## Electronic supplementary material

Figure 5A Z-stack

Supplementary Information

Supplementary Figure 7A Video

Supplementary Figure 7B Video

Supplementary Figure 8A Video

Supplementary Figure 8B Video

## Data Availability

All data generated or analysed during this study are included in this published article and its supplementary information files. The datasets generated during the current study are available from the corresponding author on reasonable request.
