# Supplementary material for: Engineered K1F bacteriophages kill intracellular Escherichia coli K1 in human epithelial cells
Source: Sci Rep. 2018 Dec 3;8:17559. doi: 10.1038/s41598-018-35859-6 (PMC6277420; doi:10.1038/s41598-018-35859-6)
Supplement: Supplementary file 2 — Supplementary Information [file 41598_2018_35859_MOESM2_ESM.pdf]

**Supplementary Information**

# Engineered K1F bacteriophages kill intracellular *Escherichia coli* K1 in human epithelial cells

Christian Moeller-Olsen[1], Siu Fung Stanley Ho[1], Ranti Dev Shukla[2], Tamas Feher[2] and Antonia P. Sagona*[1]

*Corresponding author, Antonia P. Sagona: A.Sagona@warwick.ac.uk

1. School of Life Sciences, University of Warwick, Gibbet Hill Road CV4 7AL, Coventry, UK
2. Synthetic and Systems Biology Unit, Biological Research Centre of the Hungarian Academy of Sciences, Hungary

**Table S1. Sequences of synthetic donor DNA cassettes**

| Plasmid | Donor DNA cassette |
|---|---|
| pSBGFP | gaattcgcggccgcttctagagGctgtgggtactgtgaagctgcgtgatttggcgctggaacgtgaccgtgacgtagatgct<br>cagggtgacctgattgtcggtaaatacgctatgggccacggtggtctgcgtccagaagcagcaggcgcactggttttcagcc<br>cagcggagATGCGTAAAGGCGAAGAGCTGTTCACTGGTGTCGTCCCTATTCTGGTGGAACTGGATGGTGATGTCAACGGTCA<br>TAAGTTTTCCGTGCGTGGCGAGGGTGAAGGTGACGCAACTAATGGTAAACTGACGCTGAAGTTCATCTGTACTACTGGTAAA<br>CTGCCGGTACCTTGGCCGACTCTGGTAACGACGCTGACTTATGGTGTTCAGTGCTTTGCTCGTTATCCGGACCATATGAAGC<br>AGCATGACTTCTTCAAGTCCGCCATGCCGGAAGGCTATGTGCAGGAACGCACGATTTCCTTTAAGGATGACGGCACGTACAA<br>AACGCGTGCGGAAGTGAAATTTGAAGGCGATACCCTGGTAAACCGCATTGAGCTGAAAGGCATTGACTTTAAAGAAGACGGC<br>AATATCCTGGGCCATAAGCTGGAATACAATTTTAACAGCCACAATGTTTACATCACCGCCGATAAACAAAAAAATGGCATTA<br>AAGCGAATTTTAAAATTCGCCACAACGTGGAGGATGGCAGCGTGCAGCTGGCTGATCACTACCAGCAAAACACTCCAATCGG<br>TGATGGTCCTGTTCTGCTGCCAGACAATCACTATCTGAGCACGCAAAGCGTTCTGTCTAAAGATCCGAACGAGAAACGCGAT<br>CATATGGTTCTGCTGGAGTTCGTAACCGCAGCGGGCATCACGCATGGTATGGATGAACTGTACAAATAAgcacttttagcca<br>acctaacgtcgctacagtagcggctgcacctgaagaggagactctaactcctcaacagaaagctgcgcgtactcgtgctgcg<br>aacagggccgataaactggctgagtccaacaactaattgaaaccccttgggtgcctactagtagcggccgctgcag |
| pSBN | gaattcgcggccgcttctagagaagcctgtaggactaaactatcactatagggagaccaagagatgctctgaaatgaagagac<br>tcaatgtttccctattacttcagtccatacggattgggcgtacagtaagtaataaactttatctttcaattgaataggagaat<br>tagcatATGCGTAAAGGCGAAGAGCTGTTCACTGGTGTCGTCCCTATTCTGGTGGAACTGGATGGTGATGTCAACGGTCATAA<br>GTTTTCCGTGCGTGGCGAGGGTGAAGGTGACGCAACTAATGGTAAACTGACGCTGAAGTTCATCTGTACTACTGGTAAACTGC<br>CGGTACCTTGGCCGACTCTGGTAACGACGCTGACTTATGGTGTTCAGTGCTTTGCTCGTTATCCGGACCATATGAAGCAGCAT<br>GACTTCTTCAAGTCCGCCATGCCGGAAGGCTATGTGCAGGAACGCACGATTTCCTTTAAGGATGACGGCACGTACAAAACGCG<br>TGCGGAAGTGAAATTTGAAGGCGATACCCTGGTAAACCGCATTGAGCTGAAAGGCATTGACTTTAAAGAAGACGGCAATATCC<br>TGGGCCATAAGCTGGAATACAATTTTAACAGCCACAATGTTTACATCACCGCCGATAAACAAAAAAATGGCATTAAAGCGAAT<br>TTTAAAATTCGCCACAACGTGGAGGATGGCAGCGTGCAGCTGGCTGATCACTACCAGCAAAACACTCCAATCGGTGATGGTCC<br>TGTTCTGCTGCCAGACAATCACTATCTGAGCACGCAAAGCGTTCTGTCTAAAGATCCGAACGAGAAACGCGATCATATGGTTC<br>TGCTGGAGTTCGTAACCGCAGCGGGCATCACGCATGGTATGGATGAACTGTACAAAGGcGGTGGcGGTTCTGGtGGcGGtGGc<br>TCTGGcGGTGGcGGTTCTatggcaaacgttccgggtcagaaaattggtacagaccaaggtaaaggcaaatccagctccgacgc<br>tcttgcgttgttcctgaaggtatttgccggtgaagtcctgaccgcattcactcgccgctctgtaactgctgacaagcatattg<br>tctactagtagcggccgctgcag |

| | |
|---|---|
| pSBC3 | gaattcgcggccgcttctagagggttttcagcccagcggagtaagcacttttagccaacctaacgtcgctacagtagcggctg<br>cacctgaagaggagactctaactcctcaacagaaagctgcgcgtattcgtgctgcgaacagggccgataaactggctgagtcc<br>aacaacGGcGGTGGcGGTTCTGGtGGcGGtGGcTCTGGcGGTGGcGGTTCTATGCGTAAAGGCGAAGAGCTGTTCACTGGTGT<br>CGTCCCTATTCTGGTGGAACTGGATGGTGATGTCAACGGTCATAAGTTTTCCGTGCGTGGCGAGGGTGAAGGTGACGCAACTA<br>ATGGTAAACTGACGCTGAAGTTCATCTGTACTACTGGTAAACTGCCGGTACCTTGGCCGACTCTGGTAACGACGCTGACTTAT<br>GGTGTTCAGTGCTTTGCTCGTTATCCGGACCATATGAAGCAGCATGACTTCTTCAAGTCCGCCATGCCGGAAGGCTATGTGCA<br>GGAACGCACGATTTCCTTTAAGGATGACGGCACGTACAAAACGCGTGCGGAAGTGAAATTTGAAGGCGATACCCTGGTAAACC<br>GCATTGAGCTGAAAGGCATTGACTTTAAAGAAGACGGCAATATCCTGGGCCATAAGCTGGAATACAATTTTAACAGCCACAAT<br>GTTTACATCACCGCCGATAAACAAAAAAATGGCATTAAAGCGAATTTTAAAATTCGCCACAACGTGGAGGATGGCAGCGTGCA<br>GCTGGCTGATCACTACCAGCAAAACACTCCAATCGGTGATGGTCCTGTTCTGCTGCCAGACAATCACTATCTGAGCACGCAAA<br>GCGTTCTGTCTAAAGATCCGAACGAGAAACGCGATCATATGGTTCTGCTGGAGTTCGTAACCGCAGCGGGCATCACGCATGGT<br>ATGGATGAACTGTACAAAtaattgaaaccccttgggtgccttcgggtgcttgaggggttttttgcttaaagtgagaggagactt<br>atggctcaatacattccactgaatgctaacgatgacttagatgccatcaacgatatgttagctgctatcggtgaaccagcagt<br>cctactagtagcggccgctgcag |

**Table S2. List of oligonucleotides used in this study**

| | | |
|---|---|---|
| primers used for plasmid construction: | | |
| VF2 | tgccacctgacgtctaagaa | pSB6A1 vector specific forward primer, flanking cloning site |
| VR | attaccgcctttgagtgagc | pSB6A1 vector specific reverse primer, flanking cloning site |
| GFPfw | GTTCTGCTGGAGTTCGTAACC | *gfp* gene, forward primer |
| crRNA_g10FW | AAACagcggagtaagcacttttagccaacctaacG | spacer in plasmid pCas9g10 |
| crRNA_g10Rev | AAAACgttaggttggctaaaagtgcttactccgct | spacer in plasmid pCas9g10 , reverse strand |
| g10CHECKrev | ggctaaaagtgcttactccgc | insert-specific checking primer for cloning spacer in plasmid pCas9g10 |
| crRNA_K1FC/1fw | AAACgctgagtccaacaactaattgaaaccccttG | spacer in plasmid pCas9K1FC1 |
| crRNA_K1FC/1rev | AAAACaaggggtttcaattagttgttggactcagc | spacer in plasmid pCas9K1FC1 , reverse strand |
| C1checkFW | acgctgagtccaacaactaattg | insert-specific checking primer for cloning spacer in plasmid pCas9K1FC1 |
| crRNA_K1FC/2fw | AAACgaaggcacccaaggggtttcaattagttgtG | spacer in plasmid pCas9K1FC2 |
| crRNA_K1FC/2rev | AAAACacaactaattgaaaccccttgggtgccttc | spacer in plasmid pCas9K1FC2 , reverse strand |
| C2checkFW | aaaacgaaggcacccaagg | insert-specific checking primer for cloning spacer in plasmid pCas9K1FC2 |
| pCASfor | CAGCTAGGAGGTGACTGAAG | vector-specific forward checking primer for cloning spacers in pCas9 plasmids |
| pCASrev | GGACGATCACACTACTCTTC | vector-specific reverse checking primer for cloning spacers in pCas9 plasmids |
| primers specific for K1F genome: | | |
| g11rev | GTGAAAGTGGTTGACTGAGTGG | *gene11* reverse primer |
| g10fw | GGCGACCGTTACTTCTACACC | *gene10* forward primer |
| g9fw | CAGACCGAAGCATTCAGCTCC | *gene9* forward primer |
| AS054 | CCTGATTGTCGGTAAATACGCT | *gene10* forward primer, 3’ end |

**Table S3. Recombinant phage constructs and the plasmids used to engineer them**

| construct name | phage name | donor plasmid | selection plasmid | pure clone accomplished |
|---|---|---|---|---|
| *g10::gfp* | K1F*g10::gfp* | pSBGFP | pCas9g10 | No |
| *gfp::g10* | K1F*gfp::g10* | pSBN | - | No |
| *g10b::gfp* | K1F*g10b::gfp* | pSBC3 | pCas9K1FC1, pCas9K1FC2 | Yes |

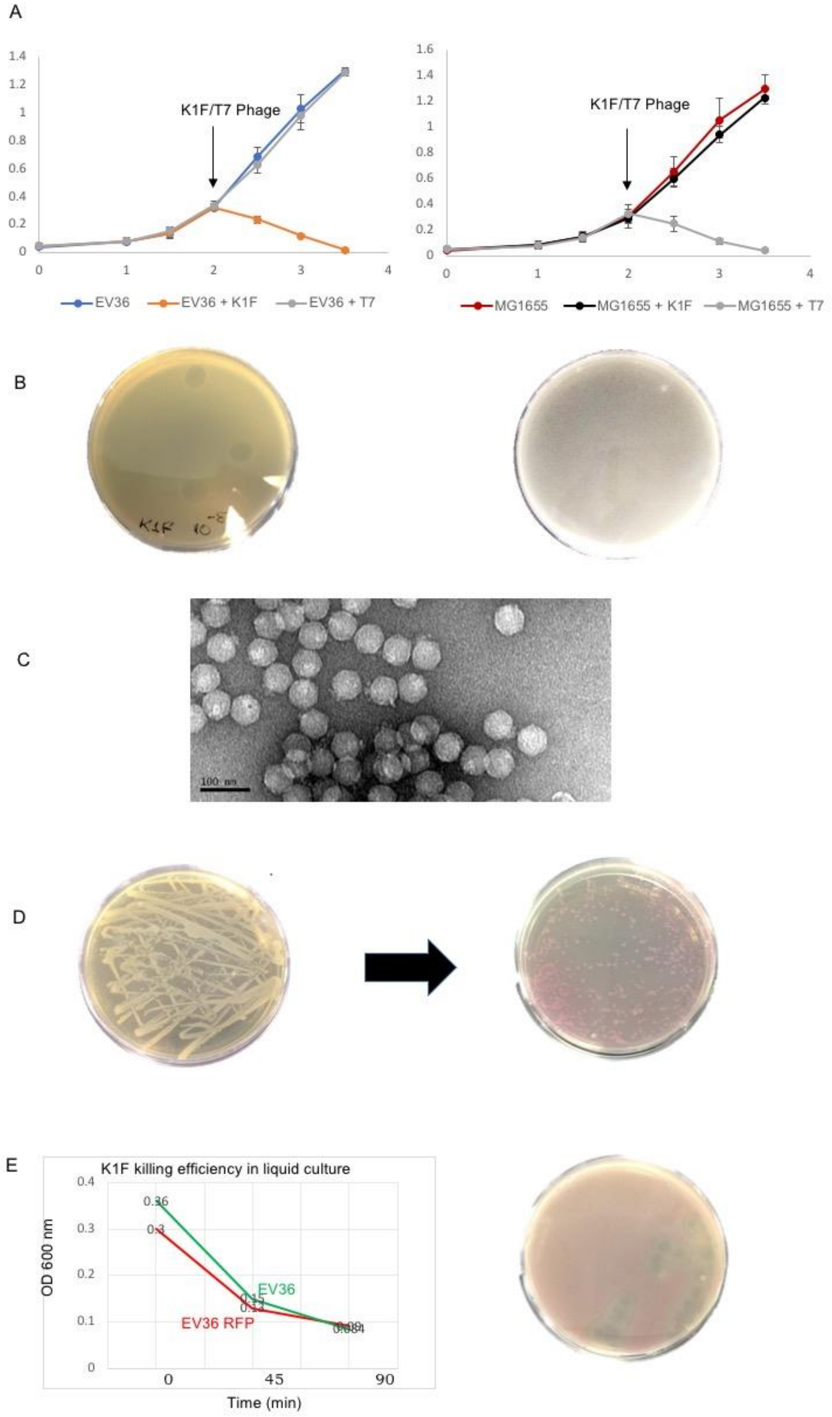


**Supplementary Figure S1.** Bacteriophage K1F is specific in targeting *E. coli* EV36. **A.** The optical density of bacterial liquid cultures was measured at 600 nm over a period of 3.5 hours. Phages were added to the *E. coli* cultures when they reached the exponential phase (2hrs) LEFT. Infection in *E. coli* EV36 liquid culture. The blue curve shows *E.coli* EV36 culture containing no phage. The orange and grey curves show cultures infected with phage K1F and

phage T7 respectively. RIGHT. Infection in *E. coli* MG1655 liquid culture. The red curve shows *E. coli* MG1655 culture containing no phage. The black and grey curves show cultures infected with phage K1F and phage T7 respectively. **B**. Clonal phage K1F was isolated from a single plaque. LEFT. Single plaques of phages K1F on a lawn of *E. coli* EV36. RIGHT. Phage K1F added onto *E. coli* MG1655 lawn and no plaques are present. **C.** Transmission electron microscopy imaging showing CsCl column purified K1F phages negatively stained with uranyl acetate. **D.** *E.coli* EV36 cells were made electrocompetent and they were transformed with a RFP plasmid (PSB6A1 from BioBrick collection) to obtain red colour and can be easily visualized by microscopy. **E.** Determination of phage K1F infection efficiency of *E.coli* EV36-RFP cells. LEFT. Optical density of liquid cultures was measured at 600 nm over a period of 90 minutes. The green and red curves show cultures of *E.coli* EV36 and *E.coli* EV36-RFP respectively. K1F phages were added to cultures at time zero. RIGHT. Plaque assay showing plaques of K1F at $10^{-7}$ dilution on a lawn of *E.coli* EV36-RFP.

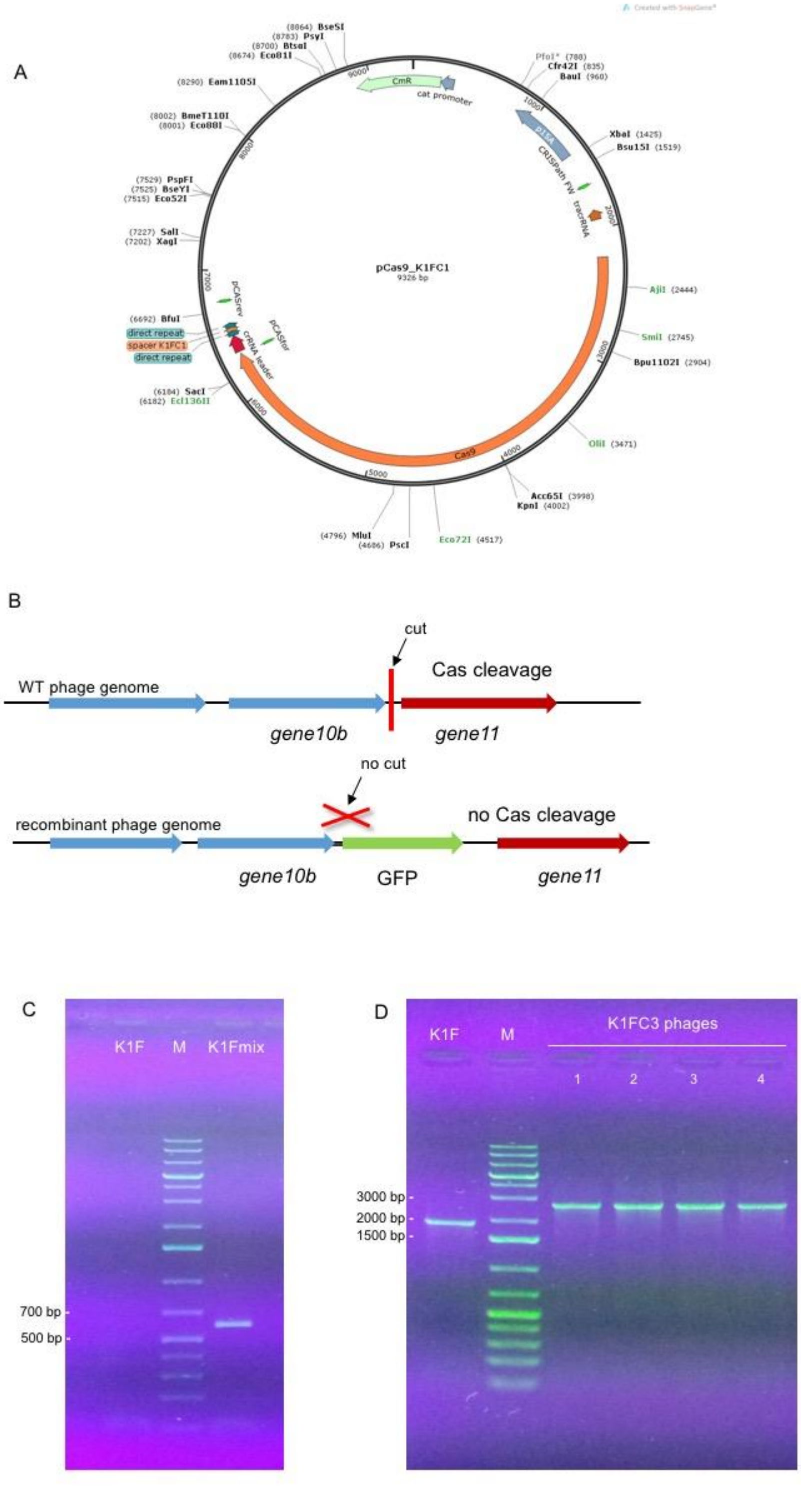


**Supplementary Figure S2.** CRISPR/Cas selection of fluorescent phage K1F. **A.** Map of plasmid pCas9K1FC1 used for CRISPR/Cas selection. Plasmid pCas9K1FC2 is identical except for the CRISPR-spacer sequence. **B.** Rationale of CRISPR/Cas selection applied on a phage mix. The crRNA of the CRISPR/Cas system is engineered such as to

cleave wild type sequences, but leave the recombinant construct intact, thereby enriching recombinant bacteriophages. **C.** The PCR product obtained with primers GFPfw and g11rev demonstrates the presence of *g10::gfp* within a phage mix obtained by phage growth on bacteria harbouring the appropriate donor plasmid. **D.** In a parallel effort to engineer *g10b::gfp*, the phage mix obtained likewise was exposed to *in vivo* CRISPR/Cas selection permitting the isolation of pure recombinant plaques displaying an elongated g9fw-g11rev PCR product, marking the integration of the GFP gene. M: GeneRuler 1 kb Plus DNA Ladder.

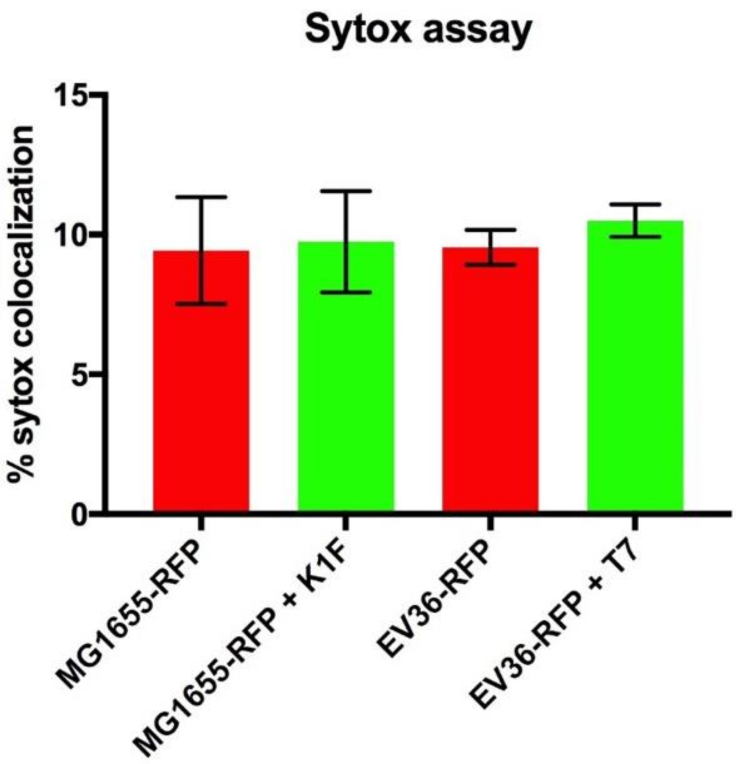


**Supplementary Figure S3.** SYTOX Bacterial Death Assay control bacteriophage-host pairs. Graph shows the average percentage of colocalisation of RFP-tagged bacteria and SYTOX DNA stain over three biological replicates +/- standard deviation. No statistical difference was observed. **LEFT.** The validity of the assay and the specificity of phage K1F was challenged using *E. coli* MG1655-RFP, a host that does not express the K1 capsid. **RIGHT.** The susceptibility of *E. coli* EV36-RFP was challenged using phage T7, a pathogen that does not naturally infect K1 expressing hosts.

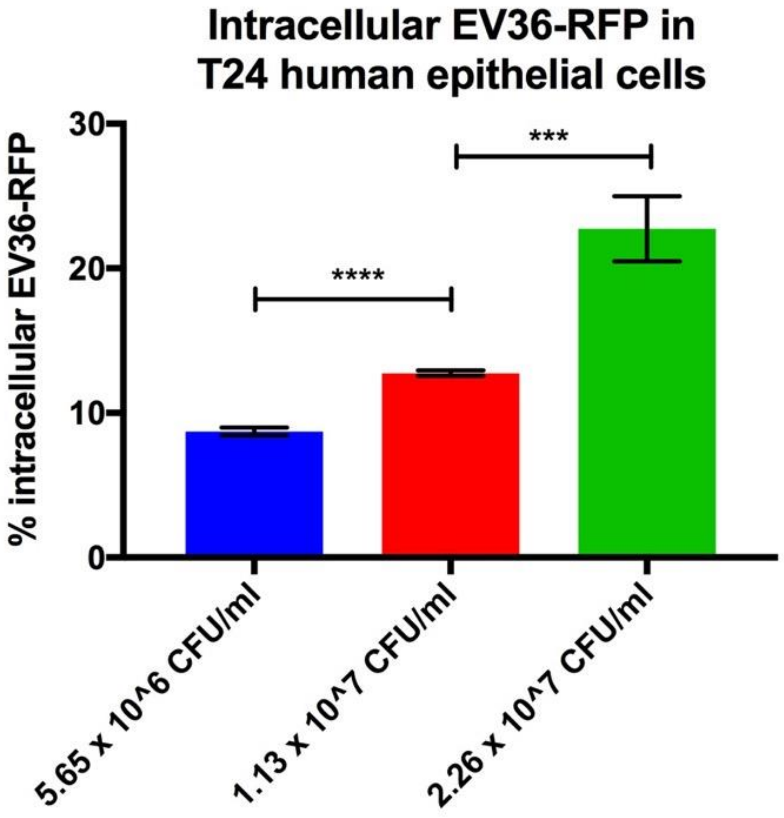


**Supplementary Figure S4.** *E. coli* EV36-RFP concentration dependent invasion in T24 cells. Human bladder epithelial T24 cells were incubated with *E. coli* EV36-RFP for two hours at final CFU/ml concentrations of 5.65 x $10^6$, 1.13 x $10^7$ and 2.26 x $10^7$. The fixed cells were imaged and prevalence of T24 cells with intracellular *E. coli* EV36-RFP was quantified as a percentage of the total T24 cell population. A representative graph of an experiment performed in biological triplicates is shown here as the average +/- standard deviation as error bars. P-values are displayed as $p \leq 0.05$ (*), $p \leq 0.01$ (**), $p \leq 0.001$ (***), $p \leq 0.0001$ (****) and not statistical significant $p \geq 0.05$ (ns). The p-value between CFU/ml 5.65 x $10^6$ and 1.13 x $10^7$ is 3.14*$10^{-5}$, and between CFU/ml 1.13 x $10^7$ and 2.26 x $10^7$ is1.56*$10^{-3}$.

A

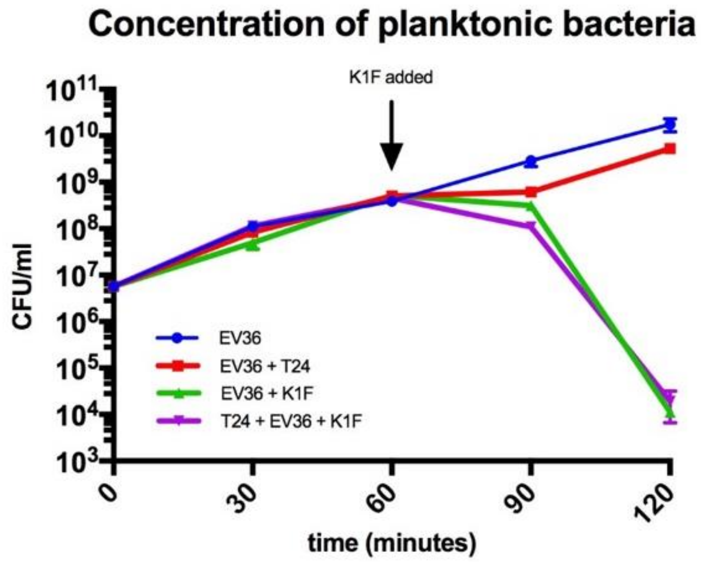


B

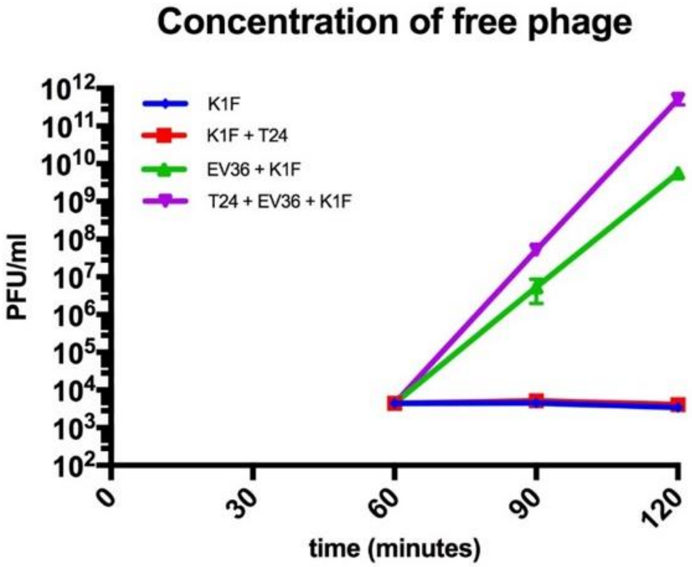


**Supplementary Figure S5.** Time course measurements of planktonic bacteria and free phages. A representative graph of an experiment performed in biological triplicates is shown here as the average +/- standard deviation as error bars. **A.** Measured concentration of planktonic *E. coli* EV36 as illustrated by CFU/ml over a two-hour time course. Bacteriophage K1F was added after one hour to corresponding wells. **B.** Measured concentration of free bacteriophage K1F as illustrated by PFU/ml over a two-hour time course.

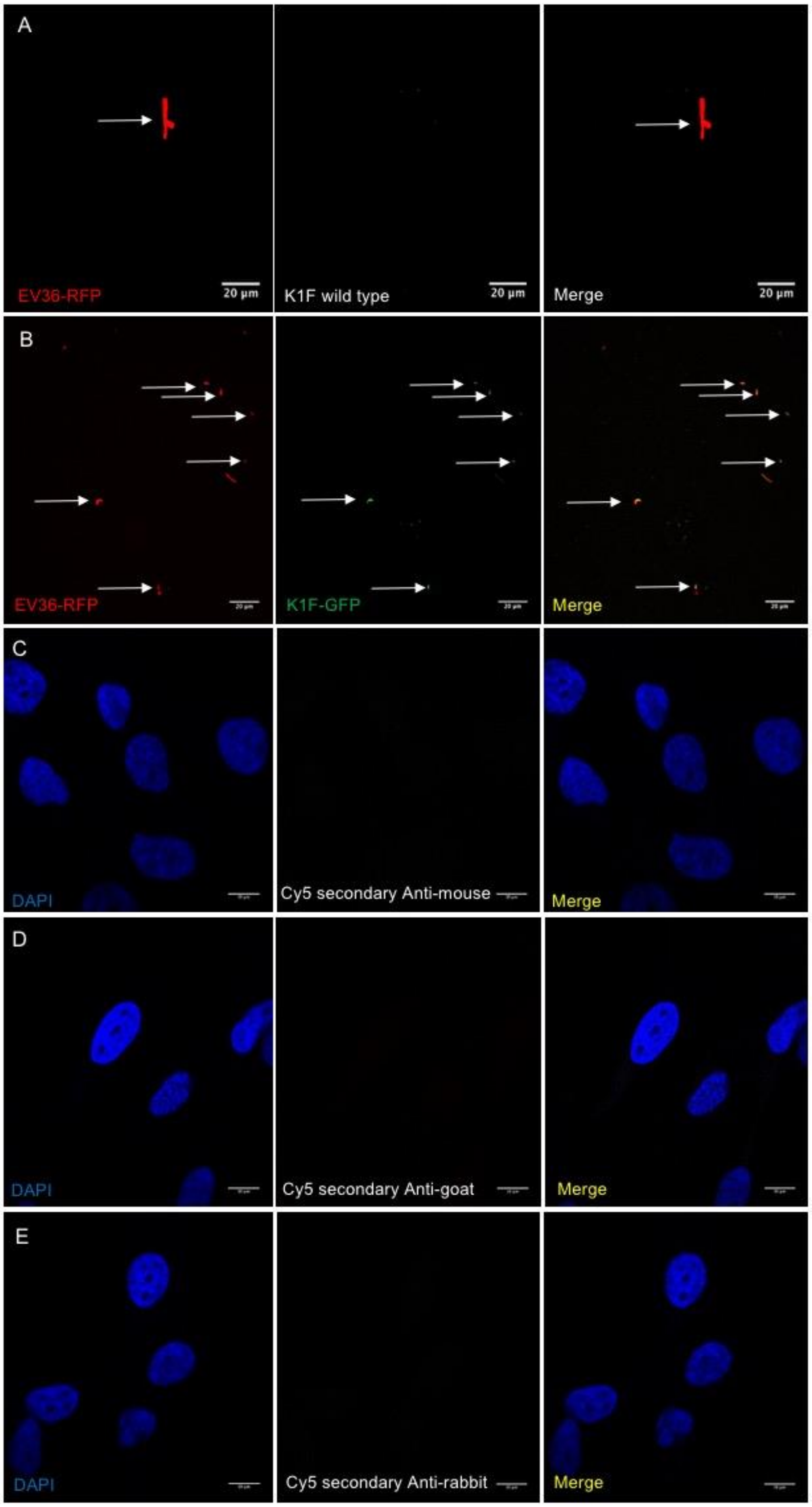
A
EV36-RFP
20 µm
K1F wild type
20 µm
Merge
20 µm
B
EV36-RFP
K1F-GFP
Merge
C
DAPI
Cy5 secondary Anti-mouse
Merge
D
DAPI
Cy5 secondary Anti-goat
Merge
E
DAPI
Cy5 secondary Anti-rabbit
Merge

**Supplementary Figure S6.** Negative controls for phage K1F-GFP signal and co-localization assays. **A-B.** Imaging of fixed T24 cells for comparison of phage K1F wild type and phage K1F-GFP. *E.coli* EV36-RFP incubated with either bacteriophage prior to fixation. (A) Showing phage K1F wild type infection of *E.coli* EV36-RFP. No fluorescent signal is observed from phage K1F wild type. **B.** Showing phage K1F-GFP infection of *E.coli* EV36-RFP, where the fluorescent signal is clearly visible (arrows). **C-E.** Negative controls for co-localization assays. Each secondary antibody used was incubated individually on fixed T24 cells and mounted with a DAPI containing medium on microscope slides. Each horizontal panel shows a representative image of each channel captured (405nm, 633nm) and merged. **C.** Goat anti-Mouse IgG (H+L) Cross-Adsorbed Secondary Antibody, Alexa Fluor 647 (Invitrogen) **D.** Cy-5 conjugated AffiniPure donkey anti-goat IgG (H+L) (Jackson ImmunoResearch) **E.** Cy-5 conjugated AffiniPure goat anti-rabbit IgG (H+L).

A

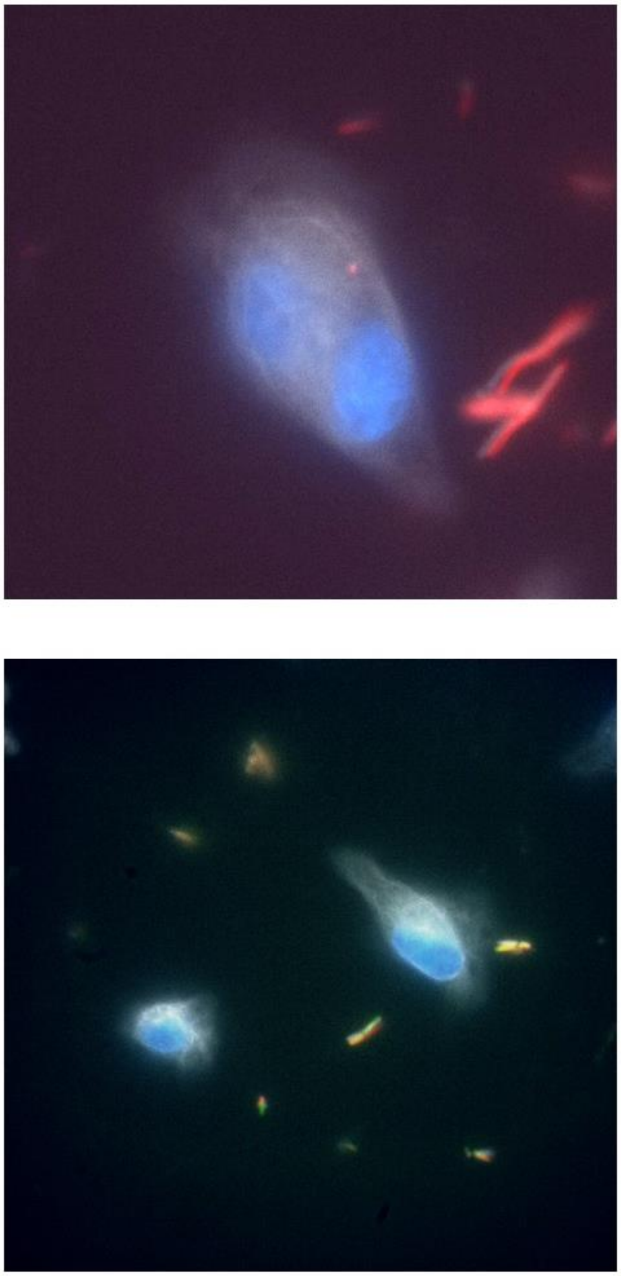

B

Supplementary Figure S7. Time-lapse live microscopy of epithelial human cells invaded by *E. coli* EV36 and incubated with fluorescent bacteriophage K1F. **A.** T24 epithelial human cells were incubated for 1 h with *E.coli* EV36-RFP bacteria grown to $OD_{600}$ of 0.2, with further filming for 1 h (pictures were taken every 10 seconds). **B.** T24 epithelial human cells were incubated for 1 h with *E.coli* EV36-RFP bacteria grown to $OD_{600}$ of 0.2. Fluorescent bacteriophage K1F was added and was incubated for 20 minutes, followed by 20 minutes filming (pictures were taken every 10 seconds). In both **A** and **B**, human cells were stained with SiR-tubulin reagent for live microtubules and NucBlue Live ReadyProbes Reagent, for the nuclear staining.

A

B

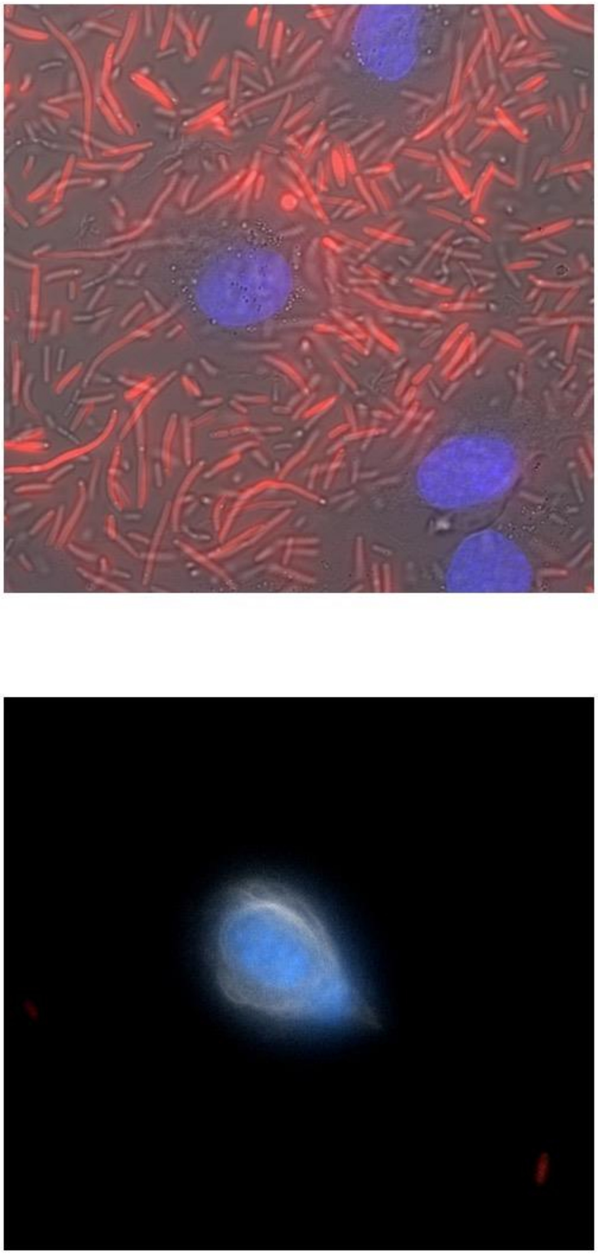

**Supplementary Figure S8.** Time-lapse live microscopy of bacteriophage K1F clearing *E. coli* EV36 bacterial infection in epithelial human cellular environment. **A.** T24 epithelial human cells were incubated for 3 h with *E.coli* EV36-RFP bacteria grown to $OD_{600}$ of 0.6, with further filming for 2 h (pictures were taken every 1 min). **B.** T24 epithelial human cells were incubated for 1 h with *E.coli* EV36-RFP bacteria grown to $OD_{600}$ of 0.2. Bacteriophage K1F was added and was incubated for 45 minutes, followed by 1 h filming (pictures were taken every 10 seconds). In both **A** and **B**, human cells were stained with NucBlue Live ReadyProbes Reagent, for the nuclear staining. In **B**, human cells were stained also with SiR-tubulin reagent for live microtubules.

**Uncropped gels**

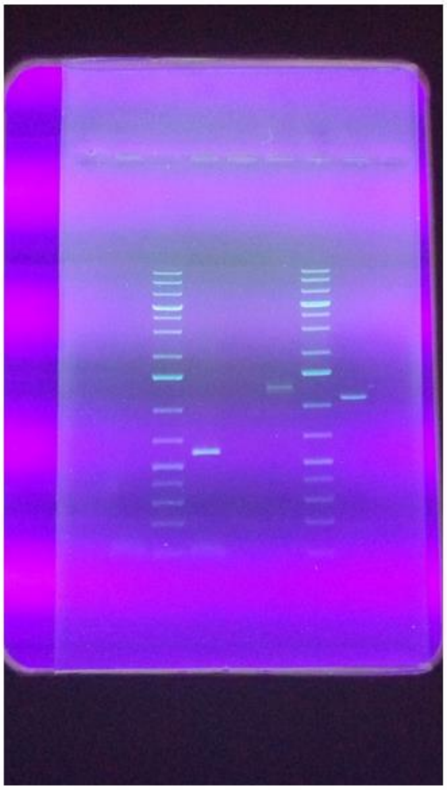

Uncropped gel Supplementary Fig. 2C

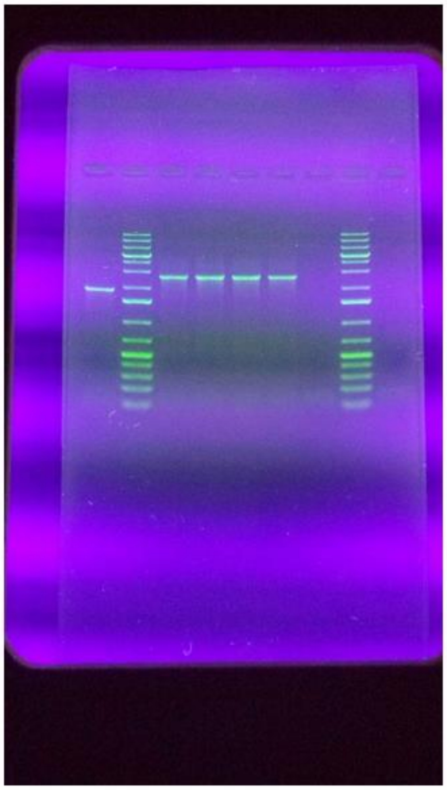

Uncropped gel Supplementary Fig. 2D
